# The Autophagy Inhibitor Spautin-1 Antagonizes Rescue of Mutant CFTR Through an Autophagy-Independent and USP13-Mediated Mechanism

**DOI:** 10.3389/fphar.2018.01464

**Published:** 2018-12-13

**Authors:** Emanuela Pesce, Elvira Sondo, Loretta Ferrera, Valeria Tomati, Emanuela Caci, Paolo Scudieri, Ilaria Musante, Mario Renda, Nesrine Baatallah, Nathalie Servel, Alexandre Hinzpeter, Diego di Bernardo, Nicoletta Pedemonte, Luis J. V. Galietta

**Affiliations:** ^1^U.O.C. Genetica Medica, IRCCS Istituto Giannina Gaslini, Genoa, Italy; ^2^Telethon Institute of Genetics and Medicine, Pozzuoli, Italy; ^3^INSERM, U1151, Institut Necker Enfants Malades, Paris, France; ^4^Université Paris Descartes, Paris, France

**Keywords:** CFTR, chloride channel, cystic fibrosis, autophagy, ubiquitination, spautin-1

## Abstract

The mutation F508del, responsible for a majority of cystic fibrosis cases, provokes the instability and misfolding of the CFTR chloride channel. Pharmacological recovery of F508del-CFTR may be obtained with small molecules called correctors. However, treatment with a single corrector *in vivo* and *in vitro* only leads to a partial rescue, a consequence of cell quality control systems that still detect F508del-CFTR as a defective protein causing its degradation. We tested the effect of spautin-1 on F508del-CFTR since it is an inhibitor of USP10 deubiquitinase and of autophagy, a target and a biological process that have been associated with cystic fibrosis and mutant CFTR. We found that short-term treatment of cells with spautin-1 downregulates the function and expression of F508del-CFTR despite the presence of corrector VX-809, a finding obtained in multiple cell models and assays. In contrast, spautin-1 was ineffective on wild type CFTR. Silencing and upregulation of USP13 (another target of spautin-1) but not of USP10, had opposite effects on F508del-CFTR expression/function. In contrast, modulation of autophagy with known activators or inhibitors did not affect F508del-CFTR. Our results identify spautin-1 as a novel chemical probe to investigate the molecular mechanisms that prevent full rescue of mutant CFTR.

## Introduction

CFTR, a plasma membrane chloride channel with a main role in epithelial cells, is mutated in cystic fibrosis (CF), one of the most frequent genetic diseases (Stoltz et al., 2015; Castellani and Assael, 2017). Loss of CFTR-dependent chloride transport affects multiple organs, including lungs, pancreas, liver, and sweat glands. Among the many types of mutations that affect the *CFTR* gene, the loss of phenylalanine 508 (F508del) is the most frequent. F508del impairs the folding and stability of CFTR protein (Lukacs and Verkman, 2012). Consequently, F508del-CFTR trafficking to the cell surface is severely altered. The mutant protein is retained in the endoplasmic reticulum and degraded by the ubiquitin-proteasome system (Lukacs and Verkman, 2012). A small fraction of the protein may reach the plasma membrane where, however, it is rapidly removed and eliminated by peripheral quality control mechanisms (Sharma et al., 2001; Okiyoneda et al., 2010; Fu et al., 2015).

Recently, pharmacological correctors of F508del defect have been developed (Galietta, 2013; Quon and Rowe, 2016). Such molecules favor F508del-CFTR trafficking with different mechanisms. One of the most advanced molecules is the corrector VX-809 (Van Goor et al., 2011). This small molecule partially rescues F508del-CFTR by possibly binding to the mutant protein itself (Ren et al., 2013; Hudson et al., 2017). VX-809 is presently used, in combination with the potentiator VX-770, to treat CF patients homozygous for the F508del mutation (Wainwright et al., 2015). However, the extent of clinical benefit obtained with the VX-809/VX-770 combination is relatively modest. The low efficacy of the combination is believed to be due to the partial activity of VX-809 as a corrector (Okiyoneda et al., 2013). A negative interaction between VX-809 and VX-770 can be also involved (Cholon et al., 2014; Veit et al., 2014). It has been shown that more marked levels of F508del-CFTR rescue can be obtained with combinations of correctors having complementary mechanisms of action (Farinha et al., 2013; Okiyoneda et al., 2013). Such other correctors may work by binding to a second site in the CFTR protein or by modulation of the cell machinery responsible for CFTR processing and degradation. Several proteins, including RNF5/RMA1, gp78, CHIP, CAL, Dab2, and cCBL, have been identified to affect CFTR processing but the list is probably far from being complete (Cheng et al., 2002; Younger et al., 2006; Morito et al., 2008; Okiyoneda et al., 2010; Ye et al., 2010; Fu et al., 2015; Tomati et al., 2015, 2018; Sondo et al., 2017). It has been shown that the ubiquitin specific peptidase 10 (USP10) is an important factor that controls CFTR degradation (Bomberger et al., 2009). Interestingly, a small molecule inhibitor of USP10, spautin-1, has been recently described. This compound also inhibits another ubiquitin peptidase, USP13 (Liu et al., 2011). By inhibiting USP10 and USP13, spautin-1 is also an inhibitor of autophagy, a process that has a possible important relationship with CFTR (Luciani et al., 2010). Therefore, we were interested in evaluating spautin-1 as a possible pharmacological tool to perturb CFTR processing. We found that spautin-1 antagonizes the rescue by VX-809 causing a rapid rundown of F508del-CFTR at the functional and molecular level. This effect may involve USP13 inhibition but is independent from autophagy block. USP13 appears as an important protein regulating the fate of mutant CFTR while spautin-1 may become an interesting probe for mechanistic studies and the search of new therapeutic agents.

## Results

**FIGURE 1:**
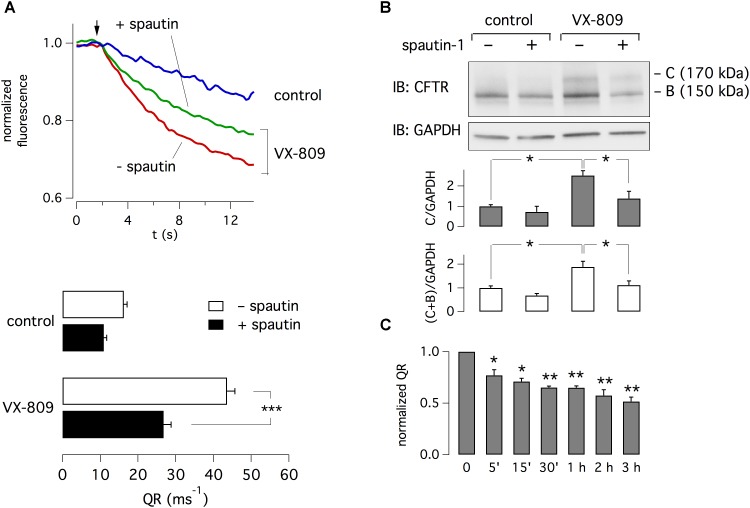
Inhibition of F508del-CFTR function and expression by spautin-1. **(A)** Representative traces and bar graphs showing F508del-CFTR activity measured with the HS-YFP microplate reader assay. The arrow indicates the time of addition of extracellular I^-^. CFBE41o- cells were treated for 24 h with vehicle (DMSO) or VX-809 (1 μM). In the last 3 h, cells also received spautin-1 (10 μM) or vehicle. Data in the graph are reported as quenching rate (QR) of HS-YFP fluorescence (*n* = 14–23 independent experiments; ^∗∗∗^*p* < 0.001). **(B)** Immunoblot analysis of F508del-CFTR expression. The image shows that both band C and band B, particularly in VX-809 treated cells, are decreased by spautin-1 (10 μM, 3 h). The bar graphs show the densitometric analysis of band C or band C plus band B, normalized for GAPDH (*n* = 3 independent experiments; ^∗^*p* < 0.05). **(C)** Time-course of spautin-1 effect. Bars report the normalized QR of HS-YFP fluorescence in cells treated with VX-809 for 24 h and then with spautin-1 (10 μM) for the indicated time (*n* = 3 independent experiments; ^∗^*p* < 0.05; ^∗∗^*p* < 0.01).

CFBE41o- cells expressing F508del-CFTR and the halide-sensitive yellow fluorescent protein (HS-YFP) were treated for 24 h with 1 μM VX-809 or vehicle (DMSO) alone. F508del-CFTR function was then determined with the HS-YFP assay in microplate reader. The treatment with VX-809 caused a nearly three-fold increase in F508del-CFTR function, as indicated by the faster fluorescence quenching caused by iodide influx (Figure 1A). We tested spautin-1 at 10 μM, the concentration previously found to affect USP10 and USP13 activity (Liu et al., 2011). When spautin-1 was added in the last 3 h of incubation, the rescue induced by VX-809 was reduced by nearly 40% (Figure 1A). The decrease in CFTR function by spautin-1 was paralleled by an altered pattern of F508del-CFTR maturation as indicated by immunoblot experiments. In lysates from untreated cells, the mutant CFTR migrates as a core-glycosylated protein (band B) with an apparent size of 150 kDa (Figure 1B and Supplementary Datasheet 2). Treatment with the corrector VX-809 is known to improve F508del-CFTR maturation with appearance of a fully glycosylated version of the protein (band C) with mobility corresponding to 170 kDa. VX-809 also increases band B abundance. Importantly, treatment with spautin-1 for 3 h antagonized the rescue by VX-809 as evident from the downregulation of band C and of total CFTR (band C plus band B; Figure 1B). Interestingly, the ratio band C/band B was not altered since both bands were downregulated by spautin-1 to a similar extent.

Using the HS-YFP functional assay, we determined the time-dependence of spautin-1 effect. Treatments with spautin-1 (10 μM) as short as 5–15 min were enough to cause a significant inhibition (20–30%) of F508del-CFTR function (Figure 1C). Larger effects were observed with longer incubations.

**FIGURE 2:**
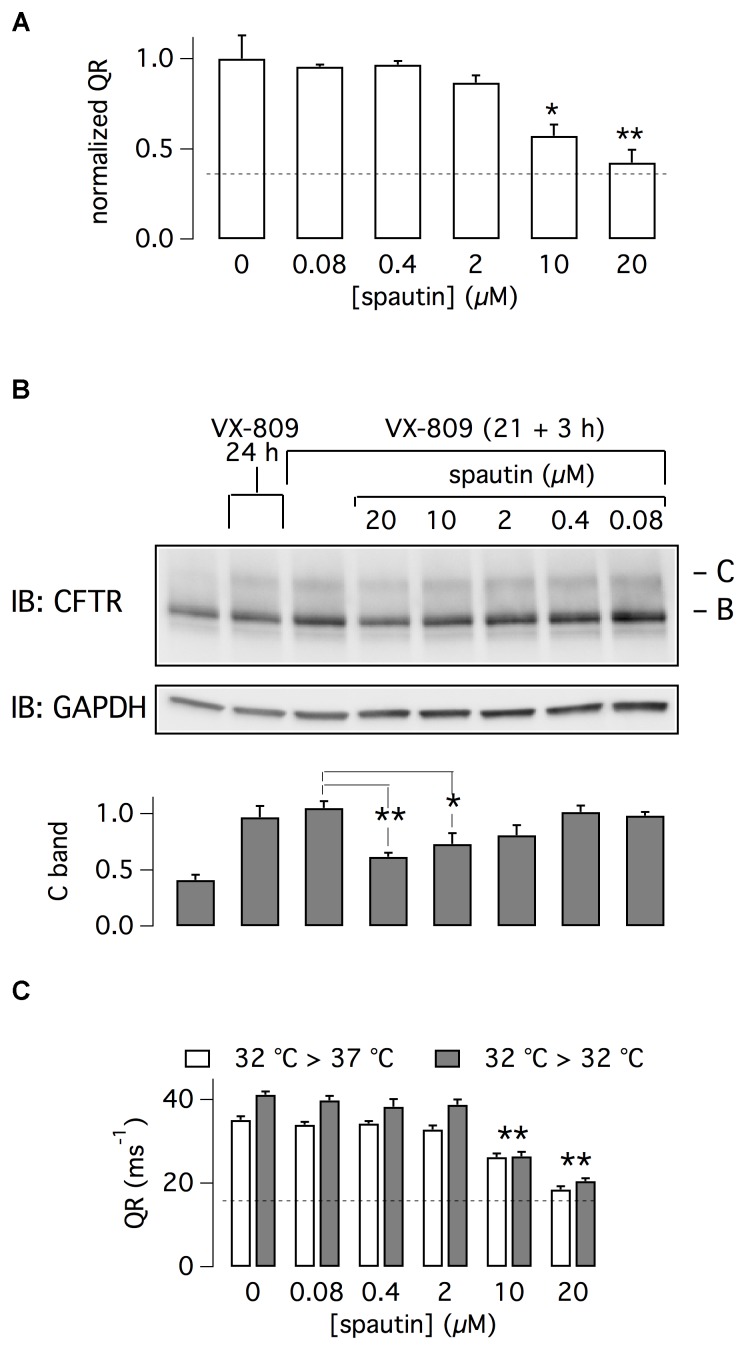
Characterization of spautin-1 effect. **(A)** Dose-response of spautin-1. The graph reports the normalized QR of HS-YFP in CFBE41o- cells treated with VX-809 (1 μM) and then, in the last 3 h, with vehicle or spautin-1 at the indicated concentrations (*n* = 5 independent experiments; ^∗^*p* < 0.05; ^∗∗^*p* < 0.01). The dashed line indicates the level of F508del-CFTR activity in uncorrected cells (no VX-809). **(B)** Immunoblot analysis of F508del-CFTR expression (representative image and densitometric analysis of band C; *n* = 3 independent experiments). CFBE41o- cells were treated with the indicated conditions (^∗^*p* < 0.05; ^∗∗^*p* < 0.01). **(C)** F508del-CFTR activity (QR) in CFBE41o- cells incubated at 32°C. In the last 3 h, cells were kept at 32°C or shifted to 37°C and treated with vehicle or the indicated concentrations of spautin-1 (*n* = 3 independent experiments; ^∗∗^*p* < 0.01 vs. vehicle).

To estimate spautin-1 potency, we tested multiple concentrations in the range 0.08–20 μM. We found that micromolar concentrations were needed to inhibit F508del-CFTR function, with 20 μM causing a nearly total reversal of VX-809 rescue (Figure 2A). A similar dose-dependence was found by examining F508del-CFTR protein maturation (Figure 2B). Importantly, at the same concentrations, spautin-1 caused no inhibition of wild type CFTR activity, irrespective of presence or absence of VX-809 (Supplementary Figure 1A). This result was consistent with unaltered pattern of CFTR protein maturation even with 20 μM of spautin-1 (Supplementary Figure 1B).

F508del-CFTR can be also rescued by cell incubation at low temperature. Cells were incubated for 21 h at 32°C, a condition that increases F508del-CFTR function as a result of enhanced expression on cell surface (Sondo et al., 2011). Then, cells were kept for three additional hours at 32°C or at 37°C, with and without spautin-1 (20 μM). As shown in Figure 2C, spautin-1 inhibited F508del-CFTR function also in cells rescued by hypothermia.

We also investigated F508del-CFTR protein expression by cell surface biotinylation. Intact cells were labeled with biotin and then lysed. Biotinylated proteins were isolated by pull-down and then revealed by immunoblot. Spautin-1 decreased F508del-CFTR but not Na^+^/K^+^-ATPase expression on cell surface (Supplementary Figure 2). It may be noted that both bands C and B are revealed by this method. The presence of core-glycosylated F508del-CFTR on cell surface, due to an unconventional route of trafficking, has already been shown in CFBE41o- cells (Gee et al., 2011; Tomati et al., 2015). In this respect, absence of calnexin and 14-3-3 proteins in the biotinylated fraction indicates that intracellular proteins were not labeled by our procedure.

**FIGURE 3:**
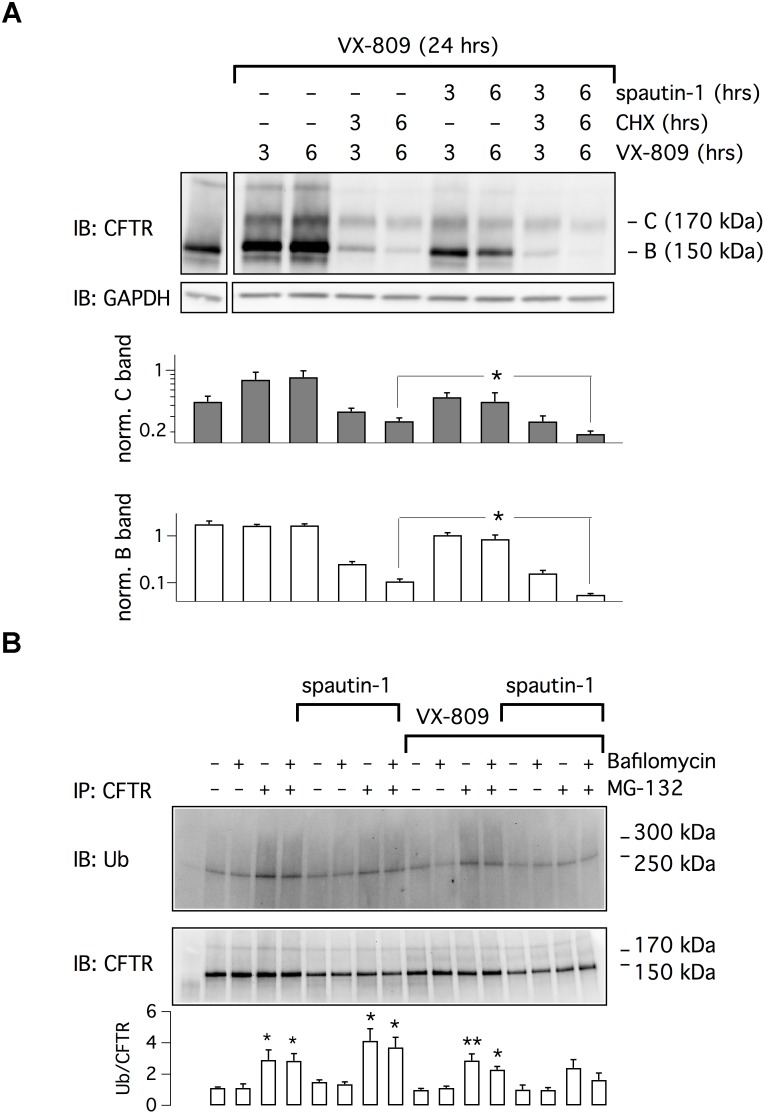
Analysis of spautin-1 mechanism of action. **(A)** Spautin-1 in the presence of protein synthesis block. The image shows a representative western blot experiment done on lysates of CFBE41o- cells treated with VX-809 (1 μM) for 24 h and, in the last 3 or 6 h, with CHX (150 μg/ml), with spautin-1 (20 μM), or with both compounds together. To apply the different conditions, the entire medium was replaced at 3 or 6 h with fresh medium containing the required compounds, including VX-809. Therefore, the times indicate for VX-809 (3 and 6 h) simply indicate the time of medium replacement (the total time of VX-809 treatment was kept at 24 h). The pattern of lysates from cells treated with vehicle alone (no corrector) for 24 h is also shown (first separate lane on the left). Bar graphs show densitometry of bands C and B, normalized for GAPDH intensity, from three independent experiments. Spautin-1 caused a significant downregulation of F508del-CFTR signal in CHX-treated cells (^∗^*p* < 0.05). **(B)** Evaluation of spautin-1 on ubiquitinated F508del-CFTR. The image shows immunodetection of CFTR and ubiquitin in cell lysates after immunoprecipitation using an anti-CFTR antibody. Where indicated, cells where treated with VX-809 (1 μM) for 24 h and, in the last 3 h, with MG-132 (10 μM), bafilomycin A1 (100 nM), and/or spautin-1 (20 μM). The image is representative of three similar experiments. The bar graph shows the ratio of ubiquitin to CFTR signals (*n* = 3 separate experiments; ^∗^*p* < 0.05; ^∗∗^*p* < 0.01 vs. corresponding condition without MG-132 and bafilomycin).

We asked whether spautin-1 effects could be due to a block in protein synthesis. CFBE41o- cells were treated with VX-809 for 24 h and, in the last 3 or 6 h, with cycloheximide (CHX) plus/minus spautin-1. In the presence of CHX and VX-809, we noted a strong reduction in both band C and band B levels (Figure 3A). However, the immature form was more markedly affected. It is interesting to note that the smear of F508del-CFTR signal, evident in cells treated with VX-809 alone, disappeared in CHX-treated cells. We interpret such results as an effect of rapid F508del-CFTR degradation (despite the presence of the corrector) that is not compensated by novel protein synthesis. Importantly, in the presence of CHX, spautin-1 was able to further decrease F508del-CFTR expression (Figure 3A).

We also investigated the effect of spautin-1 on F508del-CFTR ubiquitination (Figure 3B). After immunoprecipitation of F508del-CFTR, ubiquitin levels were assessed by immunoblot. Treatment with the proteasome inhibitor MG-132 increased the levels of ubiquitinated F508del-CFTR, also in cells corrected with VX-809. The lysosome inhibitor bafilomycin A1 was instead ineffective. Surprisingly, spautin-1 decreased the ubiquitin signal despite the presence of MG-132 or both inhibitors together (Figure 3B). This finding suggests that the F508del-CFTR disappearance elicited by spautin-1 may still occur independently of proteasome and lysosome.

**FIGURE 4:**
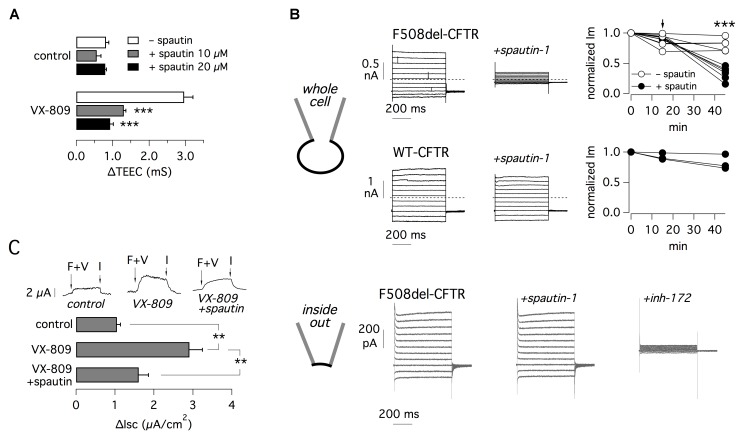
Electrophysiological evaluation of F508del-CFTR inhibition by spautin-1. **(A)** TEEC measurement in FRT cells expressing F508del-CFTR. Cells were treated with or without VX-809 (1 μM) and, in the last 3 h, with spautin-1 (10 or 20 μM) or vehicle. The graph reports the ΔTEEC value, i.e., the difference between TEEC after maximal stimulation with forskolin plus genistein and TEEC after block with PPQ-102 (see text and Supplementary Figure 3 for further details). Data are from four independent experiments (^∗∗∗^*p* < 0.001). **(B)** Results from patch-clamp experiments. The top part shows data obtained in the whole-cell configuration on FRT cells expressing F508del or wild type CFTR (representative traces and graphs reporting the normalized current measured at +100 mV). Cells expressing mutant CFTR were previously treated for 24 h with VX-809 (1 μM). During experiments, cells were acutely stimulated with forskolin (20 μM) plus genistein (50 μM). After full stimulation of mutant or wild type CFTR, the currents were measured for 15 min to check that the activity was stable. Then spautin-1 (20 μM) was added to the extracellular solution and the currents were measured for further 30–40 min. Spautin-1 elicited a significant decrease in membrane currents in F508del-CFTR but not in wild type CFTR cells. The graphs show the currents measured at the three relevant time points. Arrow shows the time of spautin-1 addition. Spautin-1 caused a significant decrease in F508del-CFTR currents (^∗∗∗^*p* < 0.001). The bottom part of panel B shows representative currents obtained in the inside-out configuration on FRT cells expressing F508del-CFTR. Currents were significantly inhibited by CFTR_inh_-172 (10 μM) but not by spautin-1 (data are representative of three similar experiments). **(C)** Effect of spautin-1 (20 μM) on human bronchial epithelial cells from a F508del-CFTR patient. Representative traces show short-circuit recordings from epithelia treated with/without VX-809 (1 μM) for 24 h. Where indicated, spautin-1 was added in the last 3 h. During recordings, F508del-CFTR was activated with 20 μM forskolin plus 1 μM VX-770 (F+V) and then blocked with 10 μM CFTR_inh_-172 (I). The graph reports the amplitude of the current drop elicited by the CFTR inhibitor (*n* = 4 separate experiments per condition; ^∗∗^*p* < 0.01).

The effect of spautin-1 was also checked on other cell types and with other assays. We studied FRT cells with expression of F508del-CFTR (Figure 4A). Such cells form tight epithelia when seeded on porous membranes (Snapwell supports). Therefore, the function of F508del-CFTR in the plasma membrane can be simply evaluated by measuring transepithelial electrical resistance (TEER), which is then converted, for convenience, to transepithelial electrical conductance, TEEC (Xue et al., 2014). Indeed, a rescue of F508del-CFTR results in higher number of chloride-conducting channels and therefore increased TEEC. It should be noted that this method measures both membranes, apical and basolateral, as two electrical resistances in series. Therefore, the detection of a drop or increase in resistance, reflecting activation or inhibition of CFTR respectively, does not distinguish whether this occurs on one or the other membrane. In this respect, it is known that CFTR traffics to both membranes in FRT cell (Sheppard et al., 1994). Therefore, TEEC changes are a reasonably good indicator of CFTR function in the plasma membrane (Xue et al., 2014).

For TEEC measurements, FRT cells were incubated for 24 h with and without VX-809 to rescue F508del-CFTR. In the last 3 h, cells were treated with or without spautin-1. At the time of the experiment, TEEC was measured under resting conditions, after maximal stimulation of F508del-CFTR with forskolin (20 μM) plus genistein (50 μM), and finally after block with PPQ-102 (30 μM), a CFTR inhibitor. Supplementary Figure 3 precisely describes the procedure. F508del-CFTR function was reported as ΔTEEC, i.e., the difference between TEEC measured with forskolin plus genistein and TEEC measured after PPQ-102 addition. Treatment with VX-809 increased ΔTEEC by more than three-fold, reflecting F508del-CFTR rescue (Figure 4A). Spautin-1 decreased the effect of VX-809 by nearly 75 and 95% at 10 and 20 μM, respectively (Figure 4A).

Effect on spautin-1 in FRT cells was also assessed using the patch-clamp technique in the whole-cell configuration (Figure 4B, top part). FRT cells expressing F508del-CFTR were treated with VX-809 (1 μM) for 24 h. During whole-cell patch-clamp recordings, F508del-CFTR was activated with forskolin (20 μM) plus genistein (50 μM). We monitored membrane currents for 15 min to check that they were stable. Then, we added spautin-1 (20 μM) by extracellular perfusion. We observed a progressive decline in F508del-CFTR function that reached a stable level (nearly 30% of original value) in 30–40 min (Figure 4B). This decline was not observed in experiments in which membrane currents were monitored for a comparable time in the absence of spautin-1 (Figure 4B). Addition of CFTR_inh_-172 (10 μM), another specific inhibitor of CFTR channel, strongly blocked the residual current (not shown). We also tested spautin-1 on cells expressing wild type CFTR (Figure 4B). In agreement with HS-YFP data, spautin-1 was largely ineffective on the normal protein (Figure 4B).

To check the dependence of spautin-1 on an intact intracellular environment, we carried out patch-clamp experiments in the inside-out configuration (Figure 4B, bottom part). After excision of the membrane patch, F508del-CFTR channels were activated by phosphorylation. Subsequent addition of spautin-1 (20 μM), kept for the duration of the recording (up to 30 min) did not significantly inhibit the current. Membrane currents were 370 ± 79 and 403 ± 113 pA before and after spautin-1 (*n* = 3), respectively. After spautin-1, block by CFTR_inh_-172 demonstrated that the recorded currents were in large part due to CFTR activity (Figure 4B, bottom).

Spautin-1 was also tested in primary bronchial epithelial cells from a F508del/F508del patient (Figure 4C). F508del-CFTR function was determined with short-circuit current recordings. The ENaC sodium channel was first blocked with amiloride (10 μM) and then F508del-CFTR was stimulated with CPT-cAMP (100 μM) and VX-770 (1 μM). Stimulation resulted in an increase in transepithelial current due to F508del-CFTR function which was subsequently blocked with CFTR_inh_-172 (10 μM). The amplitude of the current blocked by CFTR_inh_-172 indicates the amount of functional F508del-CFTR in the plasma membrane. This value increased from nearly 1 to 2.9 μA/cm^2^ after treatment for 24 h with VX-809 (1 μM). Rescue by VX-809 was decreased by 70% in cells treated for 3 h with 10 μM spautin-1 (Figure 4C).

**FIGURE 5:**
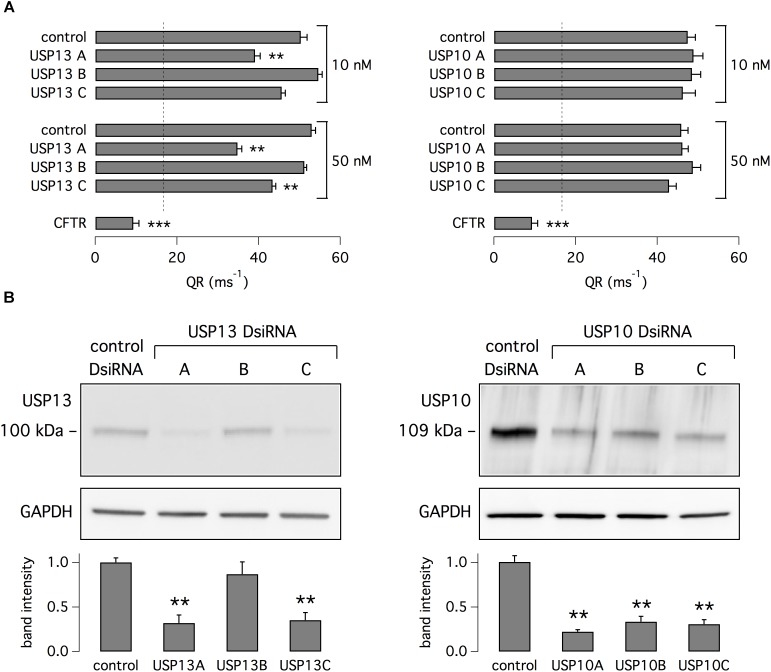
Analysis of USP13 and USP10 relationship with F508del-CFTR expression and function. **(A)** Evaluation of F508del-CFTR activity in CFBE41o- cells treated with VX-809 (1 μM). Data were obtained with the HS-YFP assay after transfection of cells with DsiRNAs (10 or 50 nM) against USP13, USP10, or CFTR. Non-silencing DsiRNA was also used as control. Dashed line reports the value of activity in cells without VX-809 treatment. Silencing of USP13 but not of USP10 significantly decreased F508del-CFTR function. Silencing of CFTR also caused a marked decrease in activity (*n* = 4–7 independent experiments; ^∗∗^*p* < 0.01; ^∗∗∗^*p* < 0.001). **(B)** Immunoblot analysis of USP13 and USP10 expression in cells transfected with indicated DsiRNAs (50 nM). Bar graphs report densitometric analysis of USP13 and USP10 bands normalized for GAPDH (*n* = 3 separate experiments; ^∗∗^*p* < 0.01 vs. control).

To elucidate the mechanism of action of spautin-1, we separately silenced USP10 and USP13, the possible targets of this small molecule. We transfected CFBE41o- cells with three different DsiRNAs against USP13 or control DsiRNA. Cells were then treated with VX-809. Two of the anti-USP13 molecules caused a significant decrease in anion transport (Figure 5A). In particular, USP13A DsiRNA decreased the corrector-dependent fraction of F508del-CFTR function by ∼50%. Importantly, the functional effect on mutant CFTR correlated with the extent of USP13 protein knockdown as revealed by western blot experiments (Figure 5B). We also transfected CFBE41o- cells with DsiRNAs against USP10. Unexpectedly, given that USP10 has been shown to control CFTR processing (Bomberger et al., 2009), USP10 knockdown (confirmed by western blot) did not affect F508del-CFTR function (Figures 5A,B). As a control, cells were also treated with siRNAs against CFTR. Anion transport was reduced by more than 80% following CFTR knockdown (Figure 5A).

**FIGURE 6:**
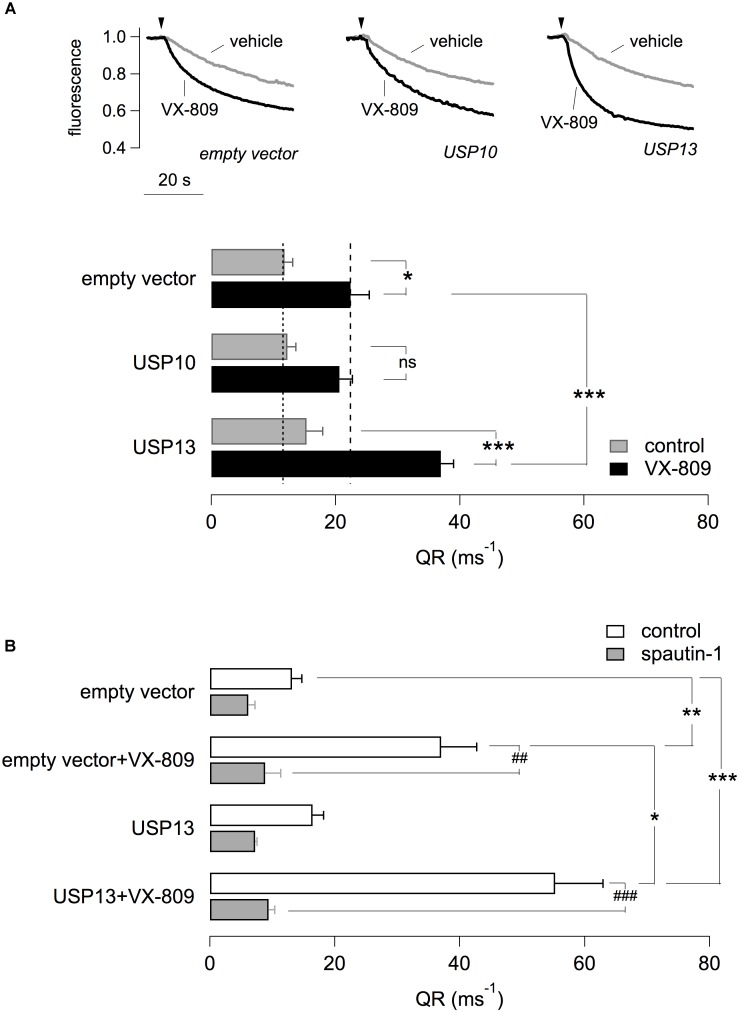
Effect of USP13 or USP10 overexpression. **(A)** Functional analysis in CFBE41o- transiently transfected with plasmids expressing USP13/USP10 or with a control plasmid. Cells also stably expressed F508del-CFTR and the HS-YFP. After transfection, cells were treated with VX-809 (1 μM) or with vehicle. The figure shows representative traces (top) and summary of QR for the different conditions (*n* = 4 independent experiments; ^∗^*p* < 0.05; ^∗∗∗^*p* < 0.001). **(B)** Effect of spautin-1 on cells transfected with USP13 and treated with/without VX-809. Data obtained with the HS-YFP assay. Spautin-1 markedly abolishes F508del-CFTR function in USP13-transfected cells (*n* = 4–7 independent experiments; ^∗^*p* < 0.05; ##/^∗∗^*p* < 0.01; ###/^∗∗∗^*p* < 0.001).

The possible role of USP13 and USP10 was also investigated by transfection in CFBE41o- cells. For this purpose, we used plasmids coding for USP13 and USP10 tagged with the mCherry red fluorescent protein. Therefore, the HS-YFP assay was carried out with a microscope and fluorescence quenching, reflecting F508del-CFTR activity, was analyzed in mCherry-positive cells. Cells were treated with VX-809 or vehicle alone. Importantly, overexpression of USP13 did not rescue F508del-CFTR by itself but significantly amplified the effect of VX-809 (Figure 6A). In contrast, overexpression of USP10 was ineffective (Figure 6A). Importantly, the effect of USP13 overexpression on F508del-CFTR function was nearly abolished by spautin-1 (Figure 6B).

**FIGURE 7:**
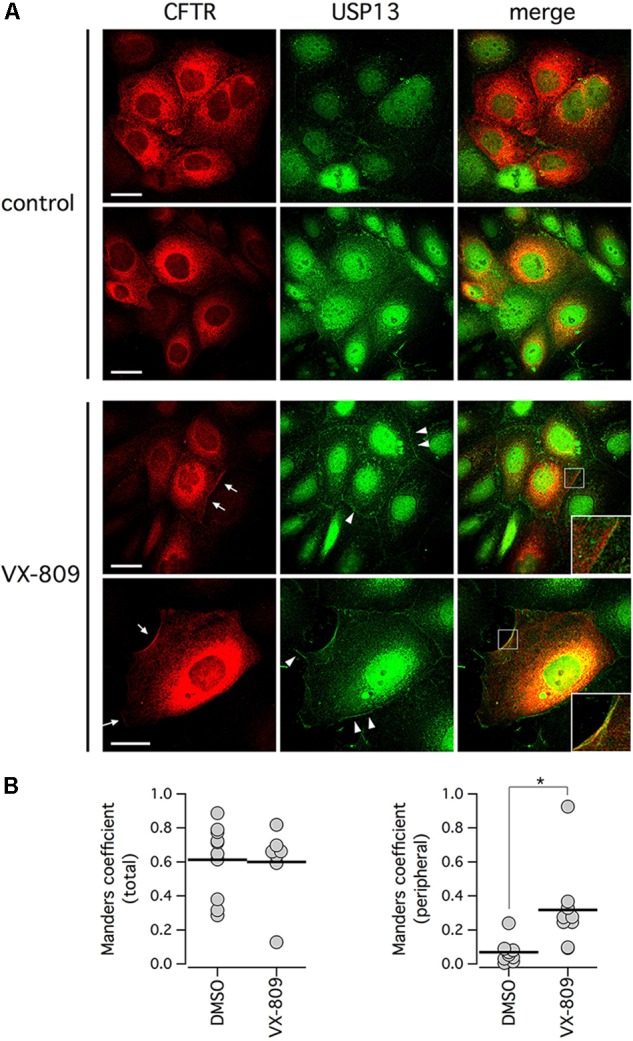
Subcellular localization of F508del-CFTR and endogenous USP13. **(A)** Confocal microscope images taken from CFBE41o- cells stained with antibodies against CFTR and USP13. Cells were treated with VX-809 (1 μM) or vehicle. Arrows indicate examples of cells with peripheral co-localization of F508del-CFTR and USP13. Arrowheads show peripheral regions with USP13 alone. Insets depict magnified cell areas of cell periphery with colocalization of USP13 and F508del-CFTR. **(B)** Colocalization analysis of F508del-CFTR and USP13 in cells treated with vehicle alone (DMSO) or with VX-809. Analysis was done on total cell area (left) or on cell periphery (right; ^∗^*p* < 0.05).

The relationship between F508del-CFTR and USP13 was further investigated by analyzing their subcellular localization with immunofluorescence. Endogenous USP13 appeared to be expressed in nucleus and cytosol (Figure 7A), in agreement with previous studies (Zhang et al., 2013; Li et al., 2017). Interestingly, cytosolic USP13 showed significant co-localization with F508del-CFTR. In particular, in cells treated with VX-809, regions of the cell periphery showing F508del-CFTR expression also showed staining for USP13 (Figure 7A, arrows). We also noticed peripheral regions with USP13 and no F508del-CFTR (Figure 7A, arrowheads) but not the opposite situation, thus suggesting that mutant CFTR at the plasma membrane or close compartments is always accompanied by USP13. The dot plots in Figure 7B show that peripheral colocalization of USP13 and F508del-CFTR is significantly increased following VX-809 treatment (Figure 7B).

**FIGURE 8:**
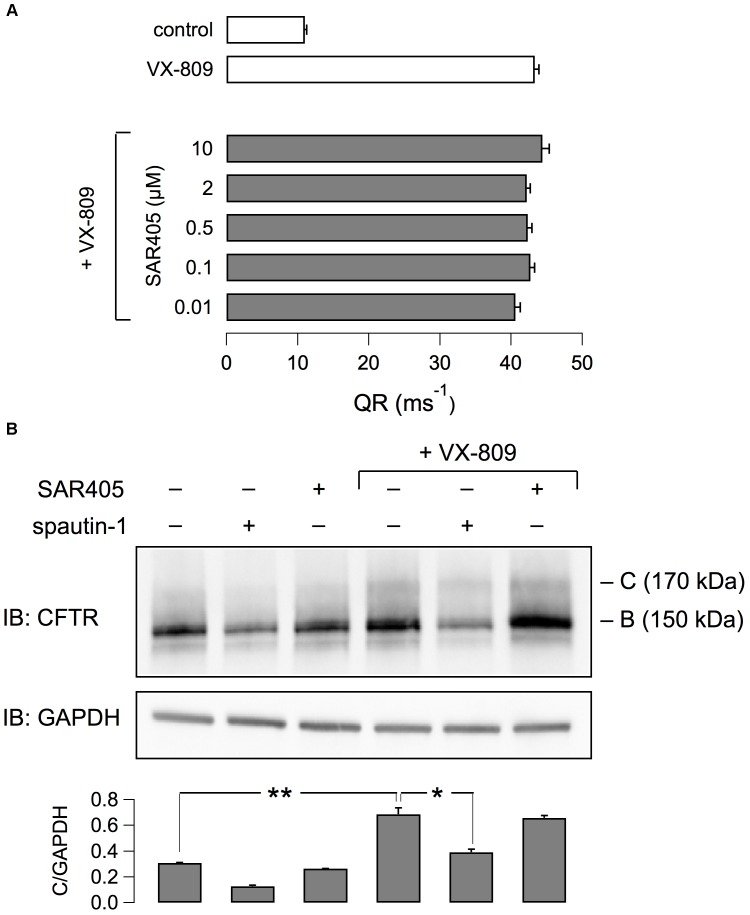
Evaluation of SAR405 on F508del-CFTR expression and function. **(A)** F508del-CFTR activity determined as QR with the HS-YFP assay. CFBE41o- cells expressing F508del-CFTR were treated with VX-809 (1 μM) and various concentrations of SAR405 or vehicle. **(B)** Immunoblot analysis of F508del-CFTR expression in cells treated (3 h) with spautin-1 or SAR405 in the presence or absence of VX-809 (1 μM, 24 h). Bar graph shows normalized densitometric analysis of band C for the different conditions (*n* = 3 independent experiments; ^∗^*p* < 0.05; ^∗∗^*p* < 0.01).

**FIGURE 9:**
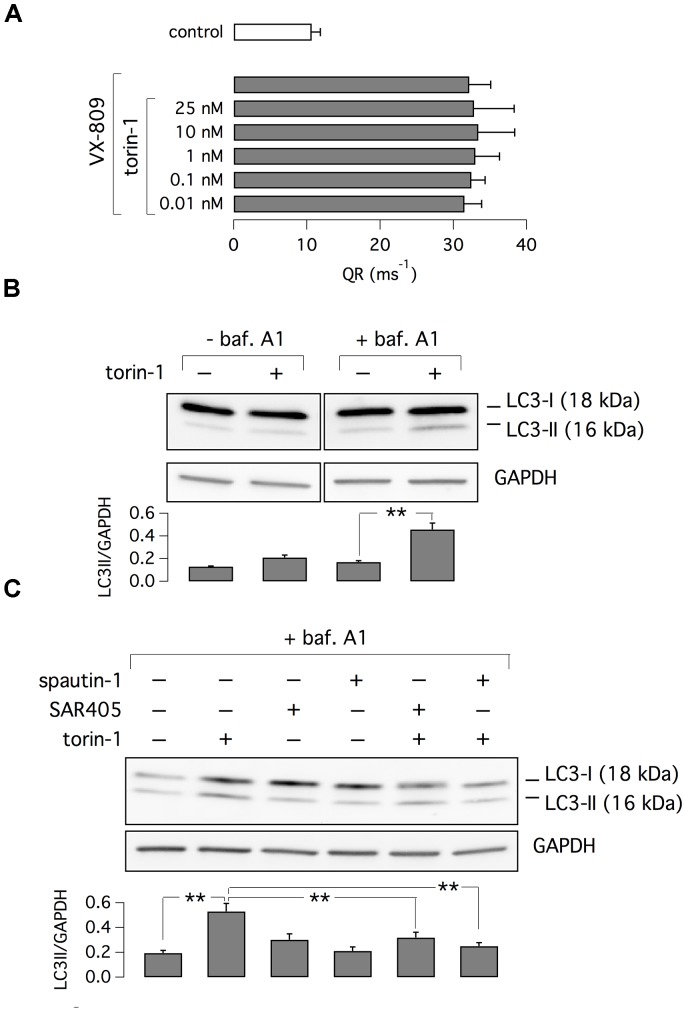
Evaluation of torin-1 as an autophagy inducer. **(A)** Test of different torin-1 concentrations on F508del-CFTR function. Cells were treated with VX-809 (1 μM) or vehicle (*n* = 3 separate experiments). **(B)** Analysis of LC3-I and LC3-II expression in cells treated with/without torin-1, to induce autophagy, and bafilomycin A1, to block lysosome-dependent degradation. **(C)** Analysis of LC3-I and LC3-II expression in cells treated with torin-1 (or vehicle) in presence/absence of SAR405 or spautin-1. Bar graphs in **(B,C)** show normalized densitometry analysis of LC3-II (*n* = 3 separate experiments; ^∗∗^*p* < 0.01).

It has been reported that spautin-1 is an autophagy inhibitor through the ubiquitin-dependent degradation of beclin-1, a subunit of the Vps34 PI3K complex (Liu et al., 2011). On the other hand, F508del-CFTR trafficking was connected to autophagy (Luciani et al., 2010). Therefore, to further investigate the mechanism of action of spautin-1 on F508del-CFTR, we also used SAR-405, another recently identified autophagy inhibitor (Ronan et al., 2014). While spautin-1 causes degradation of Vps34 complex, SAR-405 directly inhibits the catalytic activity of Vps34. Importantly, SAR-405, tested at concentrations between 10 nM and 10 μM did not affect the function of F508del-CFTR rescued by VX-809 (Figure 8A). In agreement with functional data, the expression of F508del-CFTR protein was also not affected by SAR-405 in contrast to spautin-1 (Figure 8B). To further explore the link between autophagy and F508del-CFTR, we used torin-1 as an autophagy inducer (Thoreen et al., 2009). Torin-1, tested at multiple concentrations, did not alter F508del-CFTR rescue (Figure 9A) despite being effective in increasing the expression of LC3-II, a marker of autophagy (Figure 9B). In separate experiments, we confirmed that spautin-1 and SAR-405, as autophagy inhibitors, antagonize the effect of torin-1 on LC3-II (Figure 9C).

**FIGURE 10:**
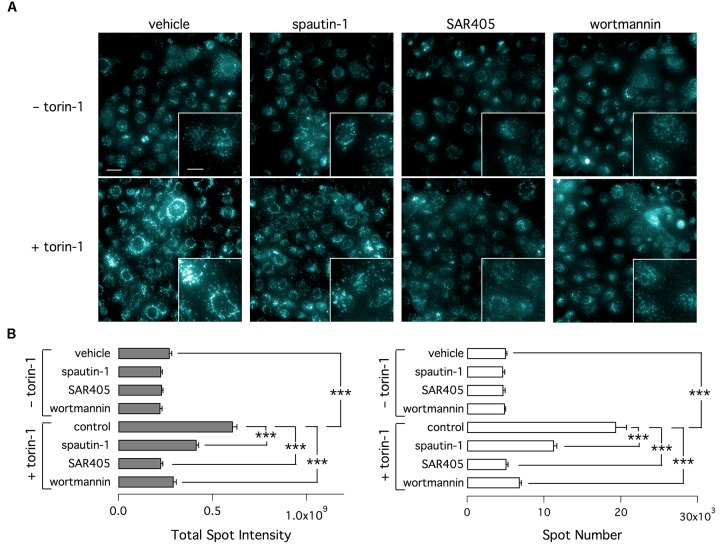
Determination of autophagy with MDC. **(A)** Representative microscopy images of CFBE41o- cells exposed to indicated treatments (torin-1, spautin-1, SAR405). Cells were stained with MDC. **(B)** Data (total spot intensity, left, and spot numer, right) obtained from MDC-stained cells (*n* = 3 separate experiments; ^∗∗∗^*p* < 0.001).

To check the effect of autophagy modulators, we also used an assay based on monodansylcadaverine (MDC). This fluorescent probe is a specific marker for autolysosomes (Biederbick et al., 1995). Indeed, MDC is accumulated inside autophagosomes. After autophagosomes fusion with lysosomes, MDC fluorescence increases due to the acidic environment (Biederbick et al., 1995; Munafó and Colombo, 2001). As shown by representative images (Figure 10A) and bar graphs (Figure 10B), torin-1 markedly increases the signal of MDC, in particular the total number and brightness of spots. This effect is significantly inhibited by spautin-1, SAR405, and wortmannin, another autophagy inhibitor acting on the Vps34 complex. Similarly to SAR405, and in contrast to spautin-1, wortmannin did not affect F508del-CFTR function (Supplementary Figure 4A). Also, the silencing of beclin 1, confirmed at the protein level, did not reduce F508del-CFTR function and expression in VX-809 treated cells (Supplementary Figures 4B–D).

In a further attempt to elucidate spautin-1 mechanism of action, we investigated the effect of dynasore, an endocytosis inhibitor. Treatment with this compound did not block the effect of spautin-1 (Supplementary Figure 5).

## Discussion

Our study reveals novel mechanisms that affect the therapeutic correction of mutant CFTR. There is a strong interest in the development of novel strategies to maximize the rescue of CFTR with the F508del mutation. This is particularly important for CF patients with a single copy of the mutation. Indeed, the drug available for clinical use, VX-809, is only modestly effective on F508del homozygotes (in combination with the potentiator VX-770) and ineffective on individuals with a single F508del allele (Rowe et al., 2017). As shown previously, F508del-CFTR, even in the presence of VX-809 or other correctors, is still intrinsically unstable and therefore recognized by quality control mechanisms and eliminated (He et al., 2013). Enhanced rescue could be obtained by combining VX-809 (or another similarly active compound) with another small molecule acting on a second site in the F508del-CFTR protein (Farinha et al., 2013; Okiyoneda et al., 2013). Alternatively, pharmacological modulation of another protein that is responsible for mutant CFTR elimination could improve the efficacy of correctors.

In the present study, we have investigated spautin-1 as a possible probe to perturb F508del-CFTR rescue. There were two reasons for testing this small molecule. First, spautin-1 is an inhibitor of USP10 (Liu et al., 2011), a deubiquitinating enzyme that is important for CFTR homeostasis (Bomberger et al., 2009). Second, spautin-1, by acting on USP10 and USP13, is also an inhibitor of autophagy (Liu et al., 2011), a cell process that has been proposed to be crucial for F508del-CFTR fate and CF pathogenesis (Luciani et al., 2010).

Short-term pre-incubation of cells with spautin-1 significantly decreased the activity of F508del-CFTR in cells treated with VX-809 or low temperature. An acute effect of spautin-1 was also observed during electrophysiological recordings of rescued F508del-CFTR activity. Importantly, we found no inhibitory effect of spautin-1 in cells expressing wild type CFTR. This finding indicates that spautin-1 is not acting as a direct blocker of CFTR or by interfering with the process of CFTR channel opening but with a mechanism that is linked with the intrinsic instability of F508del-CFTR. We also found that spautin-1 is effective in different cell types, including primary CF bronchial epithelial cells, and using different assays.

We investigated the effect of spautin-1 at the protein level. In agreement with functional data, spautin-1 altered the expression of F508del-CFTR but not of wild type CFTR. In particular, spautin-1 decreased the expression of both mature and immature forms of the mutant protein. This type of effect was also observed when the protein in the plasma membrane was detected by cell surface biotinylation. The presence of partially glycosylated F508del-CFTR (i.e., band B) in the plasma membrane is not surprising since it has been attributed to an unconventional route of protein trafficking that bypasses the Golgi (Gee et al., 2011; Tomati et al., 2015). Therefore, the decrease in F508del-CFTR function caused by spautin-1 could be explained with a decreased expression of both forms of F508del-CFTR in the plasma membrane, either by reduced trafficking to the cell surface and/or accelerated internalization. In this respect, it is particularly interesting to note the results from patch-clamp experiments. In the whole-cell configuration, spautin-1 caused a significant decrease in the activity of F508del-CFTR. This effect was not detected in inside-out patch recordings from the same cell type. Such results indicate that inhibition by spautin-1 requires an intracellular machinery that is lost upon membrane patch excision. The fast effect induced by spautin-1, with a decrease in function starting a few minutes from compound addition, is intriguing. It is possible that this initial effect is due to rapid internalization of F508del-CFTR protein. However, we could not block the effect of spautin-1 with dynasore. Therefore, if internalization is involved, it should occur through a mechanism insensitive to dynasore. Another possibility is that the initial decrease in function is caused by F508del-CFTR channel inactivation by ubiquitination at the plasma membrane.

We investigated the involvement of USP10 and USP13 in the effect of spautin-1 on mutant CFTR. We silenced USP10 and USP13 expression by transfection with specific DsiRNAs of cells that were also treated with VX-809. Surprisingly, USP10 knockdown did not result in alteration of F508del-CFTR activity. This seems to be in contrast with previous studies in which function of USP10 was found to be important in CFTR trafficking at the cell periphery (Bomberger et al., 2009). However, those studies were done on cells expressing wild type CFTR. Therefore, it is possible that USP10 is much less involved in mutant CFTR processing. Instead, we found that silencing of USP13 inhibited F508del-CFTR function. In agreement with RNAi transfection experiments, overexpression of USP13, but not of USP10, increased F508del-CFTR function above the level achieved with VX-809 incubation and this effect was blocked by spautin-1. These results indicate that USP13 is an important deubiquitinase that controls the extent of F508del-CFTR expression in the plasma membrane. Consequently, inhibition of endogenous USP13 could be the mechanism through which spautin-1 affects F508del-CFTR function and processing. It should be emphasized, however, that most small molecules are rarely absolutely specific for a single target. Therefore, we cannot exclude that spautin-1 also acts on another deubiquitinase. This issue will require further studies.

By inhibiting a deubiquitinase, spautin-1 treatment should result in increased F508del-CFTR ubiquitination, an effect that should lead to enhanced degradation. However, combined inhibition with MG-132 and bafilomycin did not prevent spautin-1 effect. In particular, MG-132 alone was sufficient to markedly enhance ubiquitination of F508del-CFTR. However, addition of spautin-1 did not further enhance ubiquitination but actually had the opposite effect. This unexpected finding could suggest that F508del-CFTR, similarly to other proteins, is also eliminated through a proteasome- and lysosome-independent pathway (Donoso et al., 2005; Mbonye et al., 2008; Lee et al., 2014). Alternatively, it should be noted that pro-collagen was found to be degraded by the lysosome with a non-conventional mechanism insensitive to bafilomycin (Omari et al., 2018). This issue needs further investigation to clarify the molecular mechanisms involved in this type of protein degradation.

We also asked whether spautin-1 affects F508del-CFTR by inhibiting autophagy. In a recently published paper, which investigated the molecular mechanisms of unconventional CFTR trafficking, spautin-1 was found to decrease F508del-CFTR in the plasma membrane (Noh et al., 2018). This effect was interpreted as an evidence of the involvement of autophagy machinery in CFTR trafficking. According to this model, autophagosomes could be used, under particular conditions, to transport immature CFTR to the plasma membrane (Noh et al., 2018). In our study, we tested two other small molecules, torin-1 (Thoreen et al., 2009) and SAR405 (Ronan et al., 2014), which act respectively as an activator and an inhibitor of autophagy. Importantly, neither SAR405 and wortmannin, as autophagy inhibitors, nor torin-1, as autophagy activator, affected F508del-CFTR in a positive or negative way. In particular, SAR405 and wortmannin act at the same early stage of autophagic process as spautin-1 (Vps34 complex). Therefore, we conclude that the effect of spautin-1 on F508del-CFTR is independent of autophagic machinery. In general, our results also indicate that pharmacological modulation of autophagy does not affect *per se* the function/trafficking of F508del-CFTR nor its rescue by VX-809.

**FIGURE 11:**
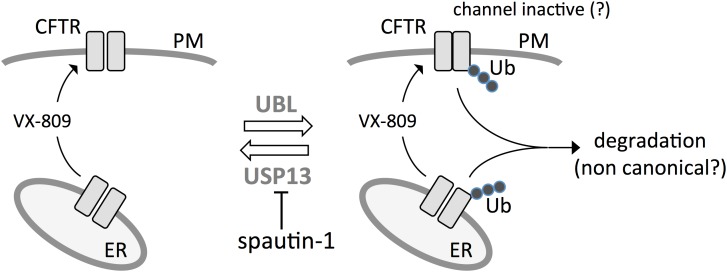
Postulated model for spautin-1 mechanism of action. F508del-CFTR is ubiquitinated at the ER and plasma membrane by ubiquitin ligases. At the plasma membrane, ubiquitination may cause internalization and, possibly, as an initial effect, also channel inactivation. Mutant CFTR is partially protected from degradation by the activity of USP13. Inhibition of USP13 with spautin-1 blocks deubiquitination thus increasing the fraction of F508del-CFTR undergoing elimination.

Summarizing, our results reveal spautin-1 as an interesting probe that works as an “anti-corrector.” In the presence of this compound, the effect of VX-809 and of another rescue maneuver, low temperature incubation, is significantly diminished. A possible explanation is that spautin-1 blocks a protective mechanism, possibly deubiquitination by USP13, which shields F508del-CFTR from degradation through a still unknown process (Figure 11). Therefore, spautin-1 can be used to reveal the underlying degradation mechanisms that limit F508del-CFTR rescue. In future studies, elucidation of spautin-1 mechanism of action may lead to the identification of novel therapeutic targets to improve rescue of F508del-CFTR and to the discovery of novel biological pathways involved in protein degradation.

## Materials and Methods

### Cell Culture Conditions

The bronchial epithelial cell line CFBE41o- with and without stable co-expression of F508del-CFTR or wild type CFTR was cultured with MEM medium supplemented with 10% fetal calf serum, 2 mM L-glutamine, 100 U/ml penicillin, and 100 μg/ml streptomycin. The generation of CFBE41o- cells co-expressing F508del-CFTR and the HS-YFP was previously described (Sondo et al., 2011). The FRT cells expressing F508del-CFTR and HS-YFP (Pedemonte et al., 2010) were cultured in Coon’s modified Ham’s F-12 medium (Sigma-Aldrich) supplemented with 10% fetal calf serum, 2 mM L-glutamine, 100 U/ml penicillin, and 100 μg/ml streptomycin.

### Fluorescence Assay for CFTR Activity (Microplate Reader Version)

CFBE41o- cells with co-expression of F508del-CFTR or wt-CFTR and the HS-YFP were treated for 24 h with DMSO or VX-809 1 μM. In some experiments, rescue of F508del-CFTR was obtained by incubation at low temperature for 24 h. Where needed, spautin-1 was added at different concentrations in the last 3 h of incubation. Shorter times of incubation with spautin-1 were also tested. After treatments, cells were washed with PBS (containing: 137 mM NaCl, 2.7 mM KCl, 8.1 mM Na_2_HPO_4_, 1.5 mM KH_2_PO_4_, 1 mM CaCl_2_, 0.5 mM MgCl_2_) and then stimulated for 30 min with forskolin (20 μM) and genistein (50 μM). Cells were then transferred to a microplate reader (FLUOstar Galaxy; BMG Labtech) for CFTR activity determination. The plate reader was equipped with high-quality excitation (HQ500/20X: 500 ± 10nm) and emission (HQ535/30M: 535 ± 15 nm) filters for YFP (Chroma Technology). Each assay consisted of a continuous 14 s fluorescence reading including 2 s before and 12 s after injection of an iodide-containing solution (PBS with Cl^-^ replaced by I^-^; final I^-^ concentration 100 mM). Data were normalized to the initial background-subtracted fluorescence. To determine I^-^ influx rate, the last 11 s of data for each well were fitted with an exponential function to extrapolate initial slope (dF/dt).

### Fluorescence Assay for CFTR Activity (Microscope Version)

CFBE41o- cells with stable co-expression of F508del-CFTR and HS-YFP, were transfected 6 h after plating with plasmids coding for USP13 or USP10 tagged with the mCherry red fluorescence protein (Origene). The day after, the cells were treated with DMSO or VX-809 (1 μM) for 24 h and, where needed, spautin-1 (20 μM) was added in the last 3 h of incubation. At the time of the assay, cells were stimulated with forskolin (20 μM) and genistein (50 μM) for 30 min and transferred to a fluorescence microscope (IX50 Olympus) equipped with a 20× objective and excitation/emission optical filters for YFP and mCherry. Images of HS-YFP fluorescence were taken with a digital camera (CoolSNAP; Photometrics) at four frames per second for a total of 40 s. At 5 s from beginning of acquisition, the high I^-^ solution was added in the well (same volumes and concentrations as indicated for the plate reader assay). A single image of mCherry-expressing cells was taken at the end of assay. For analysis of results, the MetaMorph software was used to determine the time-course of HS-YFP fluorescence quenching in mCherry-positive cells. For each well, 10 cells were considered for analysis.

### Gene Silencing With DsiRNAs

CFBE41o- cells were reverse transfected with DsiRNAs (Origene) at the time of plating. For this purpose, complexes were formed by combining DsiRNAs at the desired concentration with 0.25 μl Lipofectamine 2000 in 50 μl of Optimem synthetic medium for each well. The DsiRNA-lipofection agent complexes were pipetted in each well of a 96-well microplate together with 100 μl of MEM without antibiotics containing 50,000 cells. After 24 h of cell incubation at 37°C, the medium was removed and replaced with fresh MEM plus serum and antibiotics. After additional 24 h, the functional YFP-based assay was performed to determine the extent of F508del-CFTR activity in the plasma membrane.

### Transepithelial Electrical Conductance (TEEC)

FRT cells expressing F508del-CFTR were plated on HTS Transwell-24 well permeable supports (Code 3379, Corning Life Sciences) at a density of 200,000 cells/well. After 6 days, cells were incubated for 24 h with vehicle (DMSO) or VX-809 (1 μM) in both basolateral (800 μl) and apical (300 μl) culture medium. Where needed, spautin-1 was included in the last 3 h of treatment. After treatment, the culture medium was removed and replaced on both sides with a saline solution (100 μl apical, 800 μl basolateral) containing (in mM): 130 NaCl, 2.7 KCl, 1.5 KH_2_PO_4_, 1 CaCl_2_, 0.5 MgCl_2_, 10 glucose, 10 Na-Hepes (pH 7.4). The 24-well tray with cells was placed on a block heater (SBH 130D, Stuart) to keep the temperature at 37°C during the entire experiment. After 10 min, the basal TEER across each layer of FRT cells was measured with a STX100C electrode pair connected to an EVOM2 voltohmeter (World Precision Instruments). To stimulate F508del-CFTR, each well received (apical side) 50 μl of saline solution containing 60 μM forskolin and 150 μM genistein (final concentrations: 20 μM forskolin, 50 μM genistein). Forskolin was added to the basolateral medium to obtain the 20 μM concentration. After 10 min, TEER was measured again in each well. To block F508del-CFTR function, the inhibitor PPQ-102 was used at the final concentration of 30 μM. To achieve the desired concentration, 75 μl of the apical solution in each well was replaced with an equal volume of saline solution containing 20 μM forskolin, 50 μM genistein, and 60 μM PPQ-102. After further 10 min, the TEER was measured. All values of TEER were converted to TEEC using the formula TEEC = 1/TEER. The parameter to indicate activity of F508del-CFTR in each well, ΔTEEC was calculated from the difference in TEEC measured after maximal stimulation of F508del-CFTR with forskolin plus genistein and after block with PPQ-102 (see Supplementary Figure 3). Two particular conditions used in these experiments need to be discussed in detail. These conditions were defined during the setting of this technique. First, we used genistein instead of VX-770 as a potentiator. Indeed, the very high potency and “stickiness” of VX-770 caused problems of contamination and carry over due to adhesion to electrodes. Second, we used PPQ-102, instead of CFTR_inh_-172, to block CFTR, because the former compound is more water soluble. Therefore, it is more adequate for a procedure in which the CFTR inhibitor has to be prepared in a saline solution at three times the final concentration. For CFTR_inh_-172, this would mean 30 μM, which appeared to cause problems of solubility.

### Whole-Cell and Inside-Out Patch-Clamp Recordings

Experiments were done on FRT cells stably expressing F508del-CFTR or wild type CFTR. For whole-cell experiments, the extracellular (bath) solution had the following composition: 150 mM NaCl, 1 mM CaCl_2_, 1 mM MgCl_2_, 10 mM glucose, 10 mM mannitol, and 10 mM Na-Hepes (pH 7.4). The pipette (intracellular) solution instead contained 120 mM CsCl, 10 mM TEA-Cl, 0.5 mM EGTA, 1 mM MgCl_2_, 40 mM mannitol, 1 mM ATP, and 10 mM Cs-Hepes (pH 7.2). Acute stimulation was done by perfusion with forskolin (20 μM) plus genistein (50 μM).

For inside-out experiments, the bath solution contained: 150 mM NMDG-Cl, 2 mM MgCl_2_, 10 mM EGTA, 1 mM MgATP, 10 mM HEPES (pH 7.35). The bath solution also contained 5 μg/ml (125 nM) of protein kinase A catalytic subunit of (PKA, Promega). The pipette was instead filled with: 150 mM NMDG-Cl, 3 mM CaCl_2_, 2 mM MgCl_2_, 10 mM HEPES (pH 7.35). After excision of inside-out membrane patches, CFTR Cl^-^ channels were activated by the activity of the kinase.

During experiments, the membrane capacitance and series resistance were analogically compensated using the circuitry provided by the EPC7 patch-clamp amplifier. The usual protocol for stimulation consisted in 600 ms-long voltage steps from –100 to +100 mV in 20 mV increments starting from a holding potential of –60 mV. The waiting time between steps was 4 s. Membrane currents were filtered at 1 kHz and digitized at 5 kHz with an ITC-16 (InstruTech) AD/DA converter. Data were analyzed using the Igor software (Wavemetrics) supplemented by custom software kindly provided by Dr. Oscar Moran.

### Short-Circuit Current Recordings on Human Bronchial Epithelial Cells

Human bronchial epithelial cells obtained from a CF patient (F508del/F508del genotype) were plated on Snapwell inserts (Code 3603, Corning Life Sciences) at a density of 500,000 cells per insert. Cells were cultured for 2 weeks in a differentiating medium whose compositions has been previously described (Scudieri et al., 2012). For the first week, the medium was kept on both apical and basolateral sides of inserts (liquid–liquid condition). For the second week, the apical medium was removed (air-liquid condition, ALC). At the end of second week, cells were treated for 24 h in the basolateral medium with vehicle (DMSO) or VX-809 (1 μM). Where needed, spautin-1 was added in the medium for the last 3 h. After treatment, Snapwell inserts carrying differentiated bronchial epithelia were mounted in a vertical chamber resembling an Ussing system with internal fluid circulation. Both apical and basolateral hemichambers were filled with 5 ml of a Krebs bicarbonate solution containing (in mM): 126 NaCl, 0.38 KH_2_PO_4_, 2.13 K_2_HPO_4_, 1 MgSO_4_, 1 CaCl_2_, 24 NaHCO_3_, and 10 glucose. Both sides were continuously bubbled with a gas mixture containing 5% CO_2_ – 95% air and the temperature of the solution was kept at 37°C. The transepithelial voltage was short-circuited with a voltage-clamp (DVC-1000, World Precision Instruments) connected to the apical and basolateral chambers via Ag/AgCl electrodes and agar bridges (1 M KCl in 1% agar). The offset between voltage electrodes and the fluid resistance were canceled before experiments. The short-circuit current was recorded with a PowerLab 4/25 (ADInstruments) analogical to digital converter connected to a Macintosh computer. During recordings, cells were sequentially treated with: amiloride (10 μM, apical side) to block Na^+^ absorption through ENaC channel; CPT-cAMP (100 μM, apical and basolateral side) plus VX-770 (1 μM) to stimulate F508del-CFTR activity; CFTR_inh_-172 (10 μM, apical side) to inhibit F508del-CFTR. The difference between the current measured with CPT-cAMP plus potentiator and the current remaining after CFTR_inh_-172 activity was taken as the parameter reflecting F508del-CFTR expression in the apical membrane.

### Labeling of Autophagic Vacuoles With Monodansylcadaverine (MDC)

CFBE41o- cells stably expressing F508del-CFTR and the HS-YFP were plated (50,000 cells/well) on high quality clear-bottom 96-well black microplates suitable for high-content imaging. After 24 h, cells were treated with test compounds or DMSO (as negative control). After 24 h, cells were washed and incubated with 50 μM MDC (Sigma-Aldrich) in PBS at 37°C for 10 min (Biederbick et al., 1995). After incubation, cells were washed three times with PBS and immediately analyzed. High-content imaging and data analysis were performed using an Opera Phenix (PerkinElmer) high-content screening system. Wells were imaged in confocal mode, using a 40X water-immersion objective with high numerical aperture. MDC signal was laser excited at 405 nm and the emission was collected between 435 and 550 nm.

Data analysis of MDC signal spots was performed using the Harmony software (version 4.5) of the Opera Phenix high-content system. Briefly, for each field of view, the analysis algorithm detected the signal spots, and quantified the total number of spots, single spot intensity, and total spot intensity.

### Western Blot

Cells were grown to confluence on 60-mm diameter dishes and lysed in RIPA buffer containing a complete protease inhibitor (Roche). Cell lysates were centrifuged at 12000 rpm at 4°C for 10 min. Supernatant protein concentration was calculated using the BCA assay (Euroclone) following the manufacturer’s instructions. Equal amounts of protein (30 μg) were separated onto gradient (4–15% or 4–20% depending on target protein molecular weight) Criterion TGX Precast gels (Bio-Rad Laboratories Inc.), transferred to nitrocellulose membrane with *Trans-*Blot Turbo system (Bio-Rad Laboratories Inc.) and analyzed by Western blotting. Primary antibodies and dilutions were: mouse monoclonal anti-CFTR (596, Cystic Fibrosis Foundation Therapeutics, University of North Carolina, Chapel Hill) 1:5000; mouse monoclonal anti Na^+^/K^+^-ATPase α1 (cl. C464.6; Millipore) 1:6000; mouse monoclonal anti-GAPDH (cl.6C5; Santa Cruz Biotechnology, Inc.) 1:10000; rabbit polyclonal anti-USP13 (Abcam) 1:1000; rabbit polyclonal anti-USP10 (Abcam) 1:1000; rabbit monoclonal anti-calnexin (Abcam) 1:5000; rabbit polyclonal anti-14-3-3 epsilon (Abcam) 1:1000; rabbit polyclonal anti-LC3B (Sigma, L7543) 1:1000; rabbit polyclonal anti-Beclin-1 (Cell Signaling Technology, 3738) 1:1000. As secondary antibodies, we used anti-rabbit HRP-conjugated antibody (Abcam) diluted 1:5000 and anti-mouse HRP-conjugated secondary antibody (Ab 97023, Abcam) diluted 1:10000. Results were subsequently visualized by chemiluminescence using the SuperSignal West Femto Substrate (Thermo Scientific) and the Molecular Imager ChemiDoc XRS System. Images were analyzed with ImageJ software (National Institutes of Health). Intensity of bands was analyzed as Region-Of-Interest (ROI). The background was subtracted sand intensity was normalized against the GAPDH loading control. Data are presented as mean ± SEM of independent experiments.

### Detection of CFTR Ubiquitination

CFBE41o- cells stably expressing F508del-CFTR were grown to confluence on 60-mm diameter dishes, treated for 24 h with VX809 (1 μM) and in the last 3 h with MG132 (10 μM), bafilomicyn A1 (100nM) and/or spautin-1 (20 μM). Then, cells were rinsed twice with ice-cold Ca^2+^/Mg^2+^-free PBS and then lysed with IP Lysis Buffer (#87788 Thermo Sci.) containing EDTA-free complete protease inhibitor (Roche), N-ethylmaleimide (5 mM) and MG-132 (20 mM). Nuclei were pelleted by centrifugation at 12000 rpm at 4°C for 20 min. Supernatant protein concentration was calculated using the BCA assay (Euroclone) following the manufacturer’s instructions. An aliquot of supernatant corresponding to 500 μg of protein was incubated for 1 h with 2 μg/sample of mouse monoclonal anti-CFTR R24-1 antibody (R&D), rocking at room temperature. Antibody-antigen mixture was precipitated with 25 μl/sample of Pierce Protein A/G Magnetic Beads (Thermo Sci.) for 1 h rocking at RT, following supplier instructions. Immunoprecipitated proteins were eluted from the resin under reducing conditions with 100 μl Laemli Sample Buffer 1X, at RT. Equal amount of IP products (20 μl) were subjected to SDS-PAGE for immunoblotting analysis. Detection of CFTR and ubiquitin was performed using mouse monoclonal anti-CFTR (596, Cystic Fibrosis Foundation Therapeutics, University of North Carolina, Chapel Hill) 1:5000 and anti mouse monoclonal anti-ubiquitin (ub-P4D1, Santa Cruz Biotechnology) 1:1000. As secondary antibody, we used anti mouse-HRP-conjugated secondary antibody (Ab 97023, Abcam).

### Cell Surface Biotinylation

CFBE41o- cells were processed as previously described (Tomati et al., 2015). Briefly, cells were seeded on 100 mm dishes and treated with 1 μM VX-809 (or vehicle) for 24 h and with 20 μM spautin-1 (or vehicle) in the last 3 h. At the end of treatments, cells were washed twice with ice-cold PBS and incubated with biotin (0.35 mg/ml in PBS) for 25 min each time on a shaker at 4°C. After three washes in PBS, biotin was quenched with two washes of 50 mM NH_4_Cl in PBS (15 min each) on a shaker at 4°C. Cells were then washed three times in PBS without Ca^2+^ and Mg^2+^ and then scraped into Lysis Buffer (50 mM Hepes pH 7, 150 mM NaCl, 1% Glycerol, 1% Triton X-100, 1.5 mM MgCl_2_, 5 mM EGTA). Cell lysates were collected in an Eppendorf tube and rocked for 30 min at 4°C. Nuclei were then pelleted by centrifugation at 10,000 rpm at 4°C for 20 min. Supernatant protein concentration was calculated using the BCA assay (Euroclone) according to manufacturer’s instructions. An aliquot of supernatant corresponding to 600 μg of proteins was precipitated by rotation at 4°C for 6 h using high capacity streptavidin agarose resin (Thermo Fischer Scientific Inc.), following the manufacturer’s recommendation. The resin was then washed with the following solutions: once with Lysis Buffer, twice with Buffer 1 (150 mM NaCl, 20mM Tris-HCl, pH 8, 5 mM EDTA, 1% Triton X-100, 0.2% BSA), once with Buffer 3 (150 mM NaCl, 20 mM Tris-HCl, pH 8, 5 mM EDTA, 0.5% Triton X-100), and once with Buffer 4 (50 mM Tris–HCl, pH 8). Biotinylated proteins were eluted from the resin with reducing Sample Buffer 4X and 30 μl of each sample were separated on a 4–15% or 4–20% gradient Criterion TGX gel (Biorad) and analyzed by Western blotting.

### Immunofluorescence Detection of CFTR and USP13

CFBE41o- cells seeded in a 12-well μ-chamber (81201, Ibidi) at a density of 25.000 cells per well and treated with VX-809 and spautin-1 as for functional assays, were rinsed with PBS and fixed by adding 100 μl per well of 10% neutral buffered formalin for 5 min at room temperature. After three washes with 300 μl of PBS, cells were permeabilized with PBS-Triton X-100 0.3% for 5 min, blocked with PBS-BSA 1% for 2 h, and then incubated overnight at 4°C with 100 μl of primary antibodies diluted in blocking solution. The following antibodies and dilutions were used: ab570 mouse IgG1 anti-CFTR (J. R. Riordan, University of North Carolina at Chapel Hill, and Cystic Fibrosis Foundation Therapeutics) at 1:250 and ab99421 rabbit anti-USP13 (Abcam) at 1:200. Following incubation with primary antibodies, cells were rinsed three times with PBS and incubated with 100 μl of a solution of secondary goat anti-rabbit Alexa Fluor-488 and goat anti-mouse Alexa Fluor-546 antibodies (Invitrogen) diluted in PBS-BSA 1% for 1 h in the dark. After three further washes in PBS, cells were covered with mounting medium and coverslip, and then analyzed using a laser-scanning confocal microscope SPE (Leica Microsystems). Image analysis was performed using Leica and ImageJ software. Fractional overlap between CFTR and USP13 was quantified via Manders’ colocalization coefficient in total cell ROIs or in peripheral ROIs by using Coloc2 plugin.

### Statistics

Data are shown as representative traces/images or as a mean values and SEM of independent experiments. Significance of differences was established with ANOVA (with Tukey or Dunnett *post hoc* tests, as appropriate) except for experiments shown in Figures 4B, 7B in which Student’s *t*-test was used. Analysis was done with InStat (GraphPad) software.

## Ethics Statement

The protocols to isolate, culture, store, and study bronchial epithelial cells from patients undergoing lung transplant (described in Scudieri et al., 2012) were approved by the Regional Ethical Committee (Comitato Etico Regionale) under the supervision of the Italian Ministry of Health (registration number: ANTECER, 042-09/07/2018). Informed and written informed consent was obtained from all patients using a form that was also approved by the same Ethical Committee.

## Author Contributions

EP, ES, NP, AH, and LG conceived the study. EP, ES, LF, VT, EC, PS, IM, MR, NB, NS, DdB, and NP investigated and validated the data. EP, ES, NP, AH, and LG wrote the manuscript. NP and LG acquired the funding. NP, DB, AH, and LG provided the resources.

## Conflict of Interest Statement

The authors declare that the research was conducted in the absence of any commercial or financial relationships that could be construed as a potential conflict of interest.
