# Supplementary material for: The Autophagy Inhibitor Spautin-1 Antagonizes Rescue of Mutant CFTR Through an Autophagy-Independent and USP13-Mediated Mechanism
Source: Front Pharmacol. 2018 Dec 13;9:1464. doi: 10.3389/fphar.2018.01464 (PMC6300570; doi:10.3389/fphar.2018.01464)
Supplement: Supplementary file 1 [file Data_Sheet_1.pdf]

Supplementary Figures

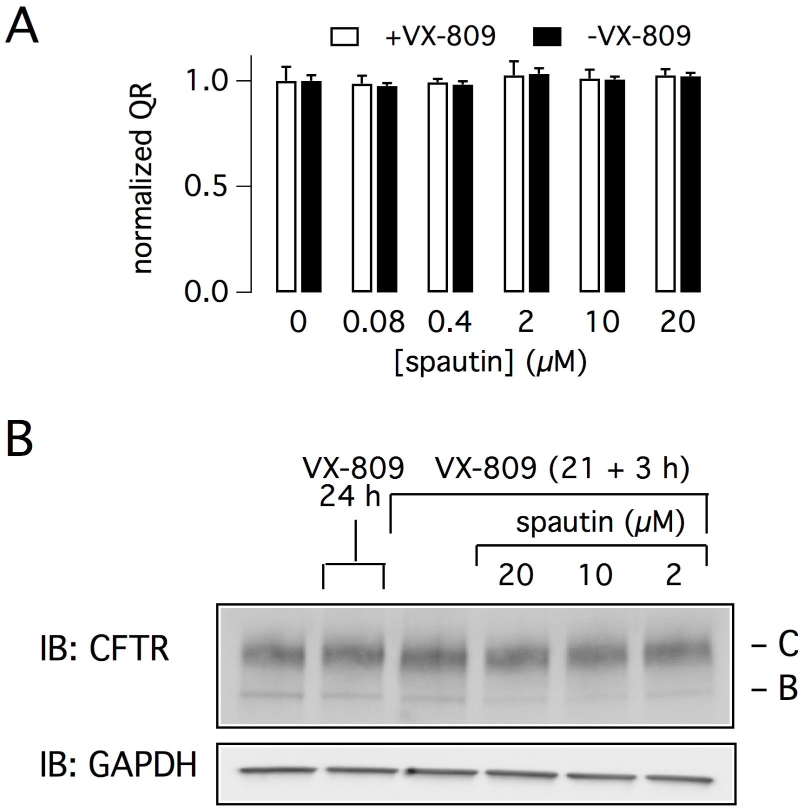

Supplementary Figure 1. Test of spautin-1 on wild type CFTR. (A) Results from the HS-YFP assay (QR values) obtained in CFBE41o- cells expressing wild type CFTR. Cells were incubated with or without VX-809 (1 μM) for 24 hours and in the last three hours with vehicle or spautin-1 at the indicated concentrations (n = 3 independent experiments). (B) Immunoblot analysis of wild type CFTR expression in cells treated with VX-809 and, where indicated, with different concentrations of spautin-1. The results are representative of three independent experiments.

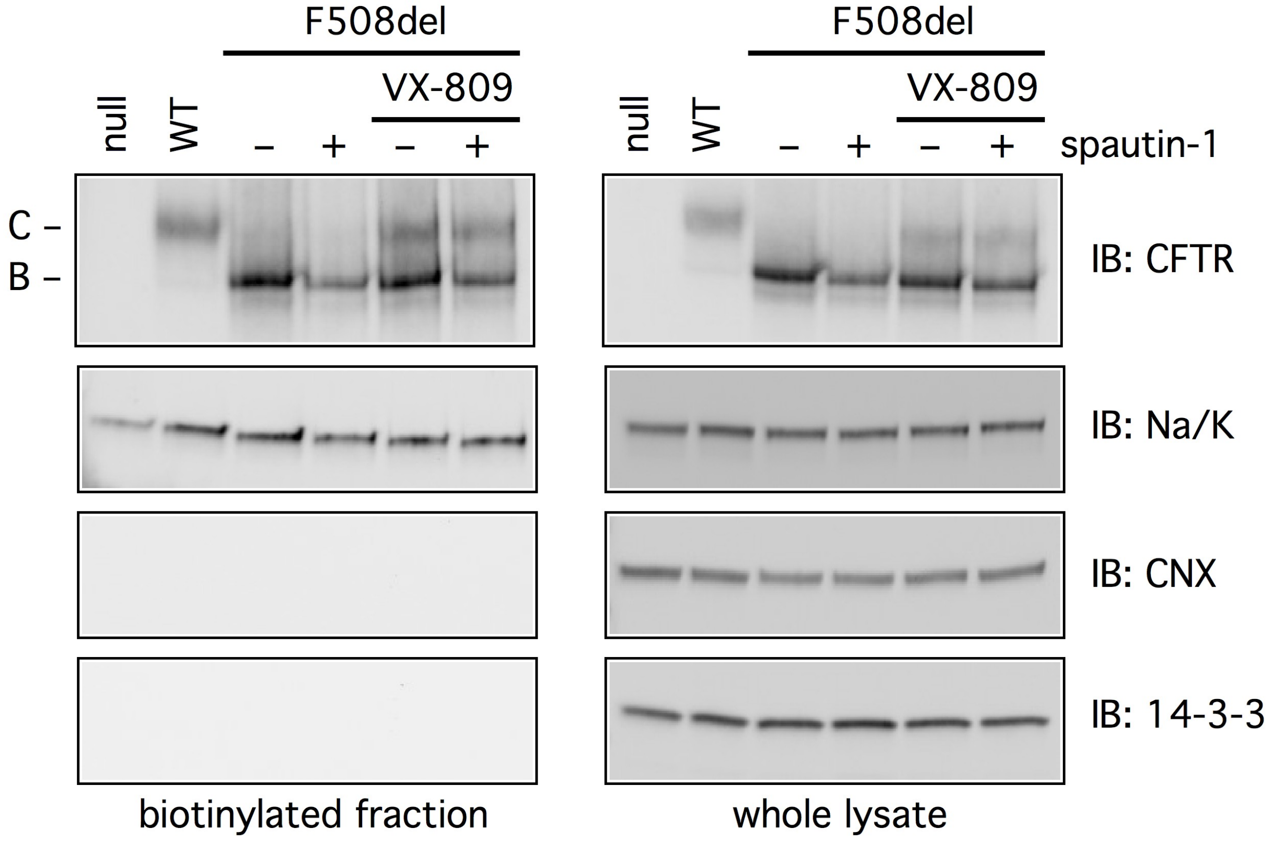

Supplementary Figure 2. Effect of spautin-1 on cell surface F508del-CFTR. Cell surface biotinylation allowed analysis of F508del-CFTR expression in the plasma membrane (left). VX-809 (1 µM) and spautin-1 (20 µM) increased and decreased the levels of band C and B, respectively. Na^{+}/K^{+}-ATPase was also present in the biotinylated fraction but its expression was not affected by spautin-1. As expected, the intracellular proteins calnexin and 14-3-3ε were present in whole lysates but not in the biotinylated fraction. Data shown in the figure are representative of three independent experiments.

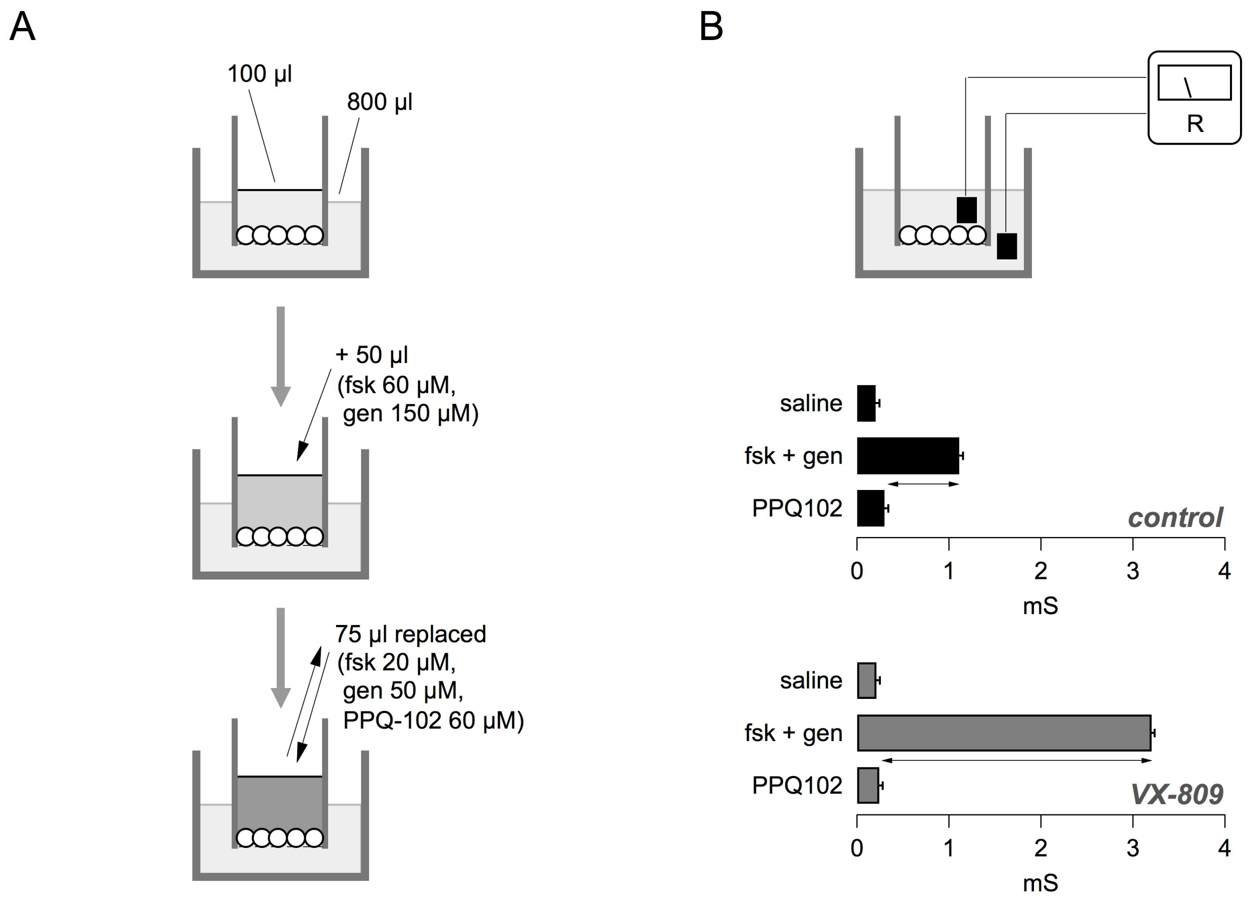

Supplementary Figure 3. Measurement of F508del-CFTR function with the transepithelial electrical conductance (TEEC) technique. (A) Protocol for stimulation and block of F508del-CFTR using forskolin/genistein and PPQ-102, respectively (see Methods for further details). (B) Scheme of transepithelial resistance measurement and examples of TEEC values obtained in FRT cells treated with vehicle or VX-809 (1 µM). ΔTEEC was the parameter considered to reflect F508del-CFTR function.

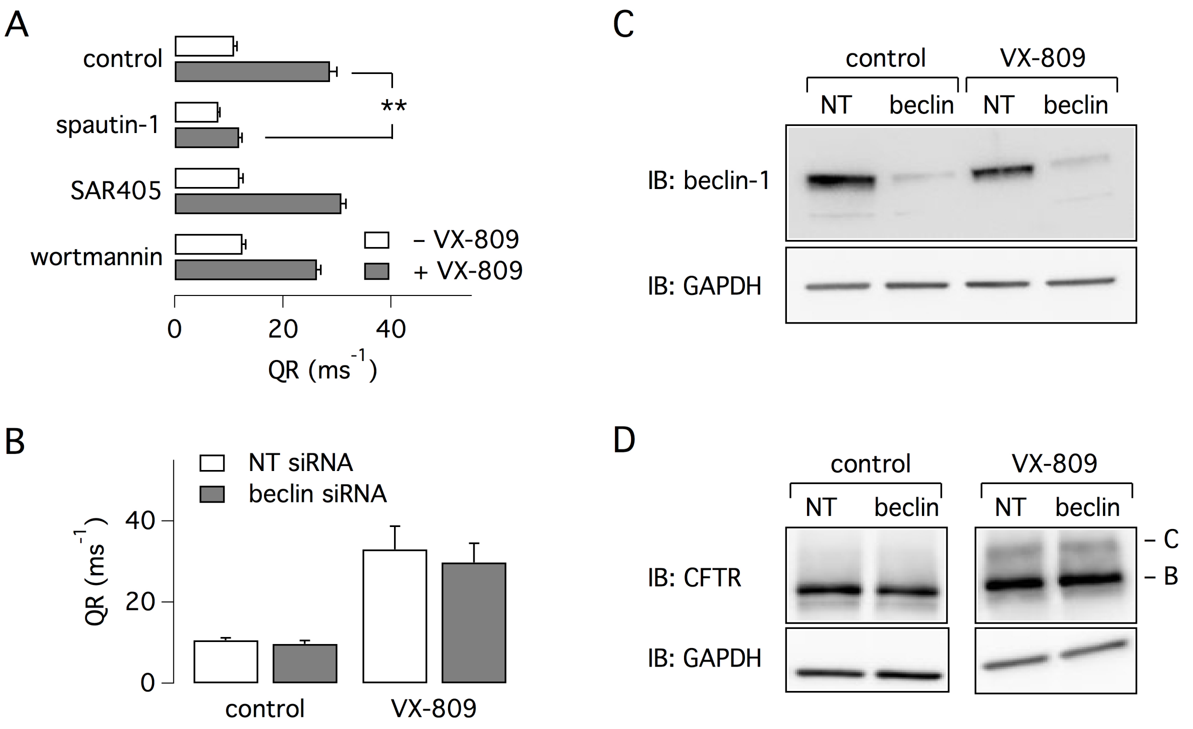

Supplementary Figure 4. Inhibition of autophagy and F508del-CFTR function. (A) Activity of F508del-CFTR (HS-YFP assay) in CFBE41o- cells incubated for 24 hours with or without VX-809 (1 µM). In the last three hours, cells were treated with the indicated compounds. Each bar is the mean ± SEM (n = 3); **, $p < 0.01$. (B) Activity of F508del-CFTR (HS-YFP assay) in CFBE41o- cells transfected with siRNA against beclin 1 or with non-targeting (NT) siRNA. Cells were also treated with or without VX-809. Beclin 1 knockdown did not affect F508del-CFTR function. (C) Western blot experiment demonstrating effective knockdown of beclin 1 by siRNA transfection. Results shown for beclin knockdown are representative of six experiments. (D) Analysis of F508del-CFTR expression in cells with beclin 1 knockdown, with and without VX-809. The images are representative of three separate experiments.

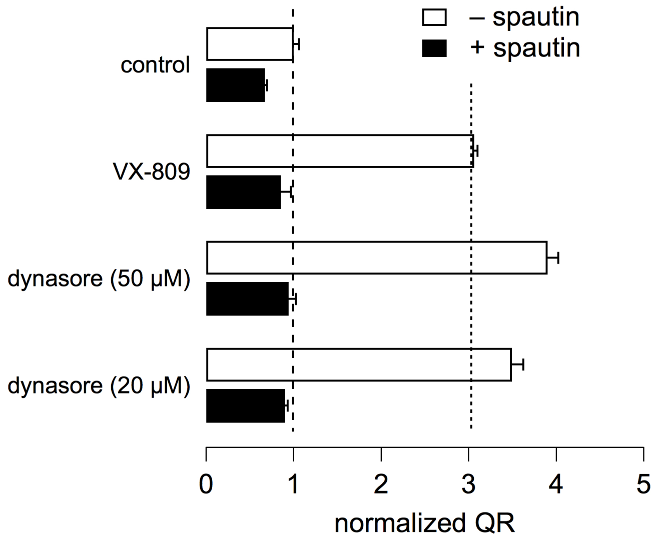

Supplementary Figure 5. Evaluation of dynasore effect. The graph shows QR values (HS-YFP assay) obtained from CFBE41o- cells expressing F508del-CFTR. Cells were treated for 24 hours with VX-809 (1 µM) or vehicle and, where indicated, in the last three hours, with spautin-1 (20 µM) and/or dynasore (20-50 µM). Data are from 4 independent experiments.
