# Supplementary material for: The Autophagy Inhibitor Spautin-1 Antagonizes Rescue of Mutant CFTR Through an Autophagy-Independent and USP13-Mediated Mechanism
Source: Front Pharmacol. 2018 Dec 13;9:1464. doi: 10.3389/fphar.2018.01464 (PMC6300570; doi:10.3389/fphar.2018.01464)

Pesce et al.

The autophagy inhibitor spautin-1 antagonizes rescue of mutant CFTR through an autophagy-independent and USP13-mediated mechanism

UNCROPPED IMMUNOBLOT IMAGES

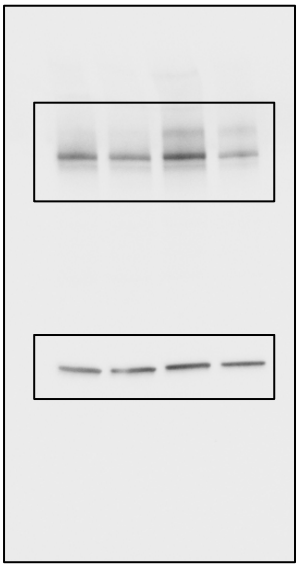

Figure 1B

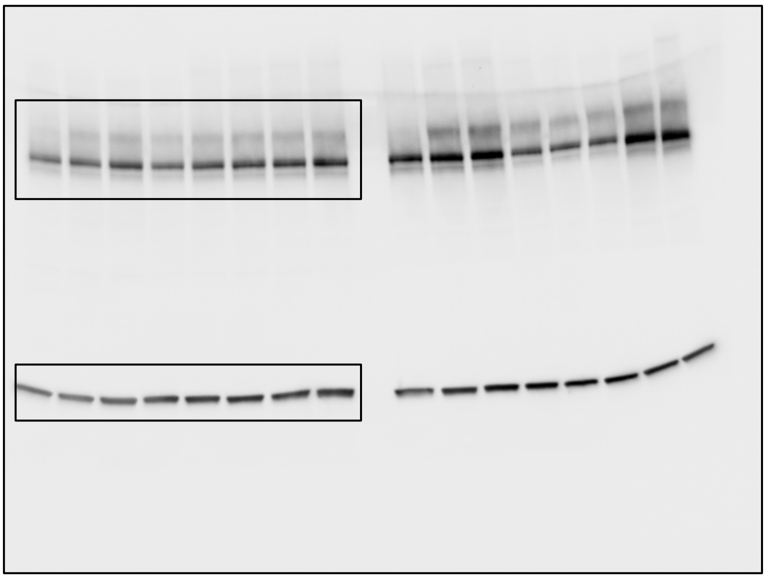

Figure 2B

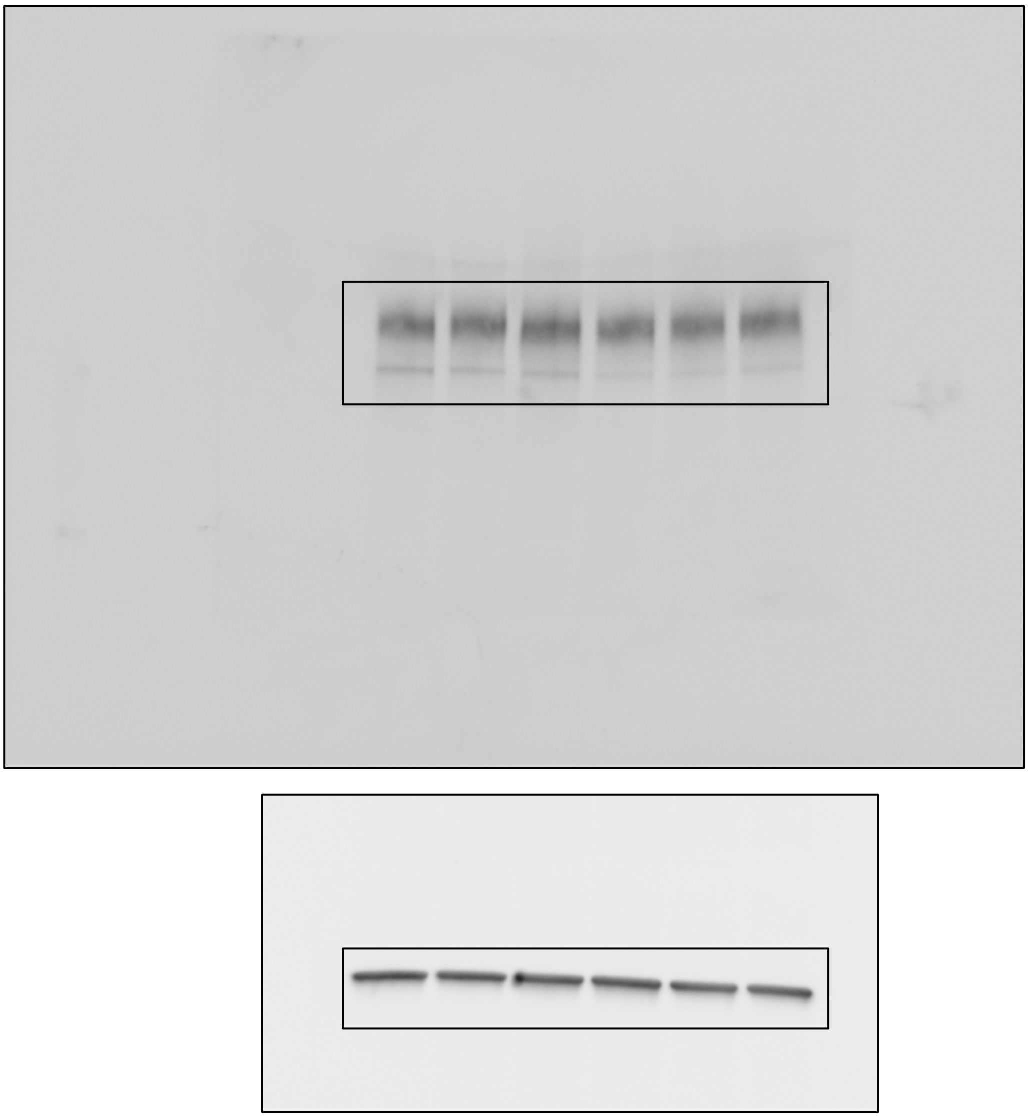

Figure 2D

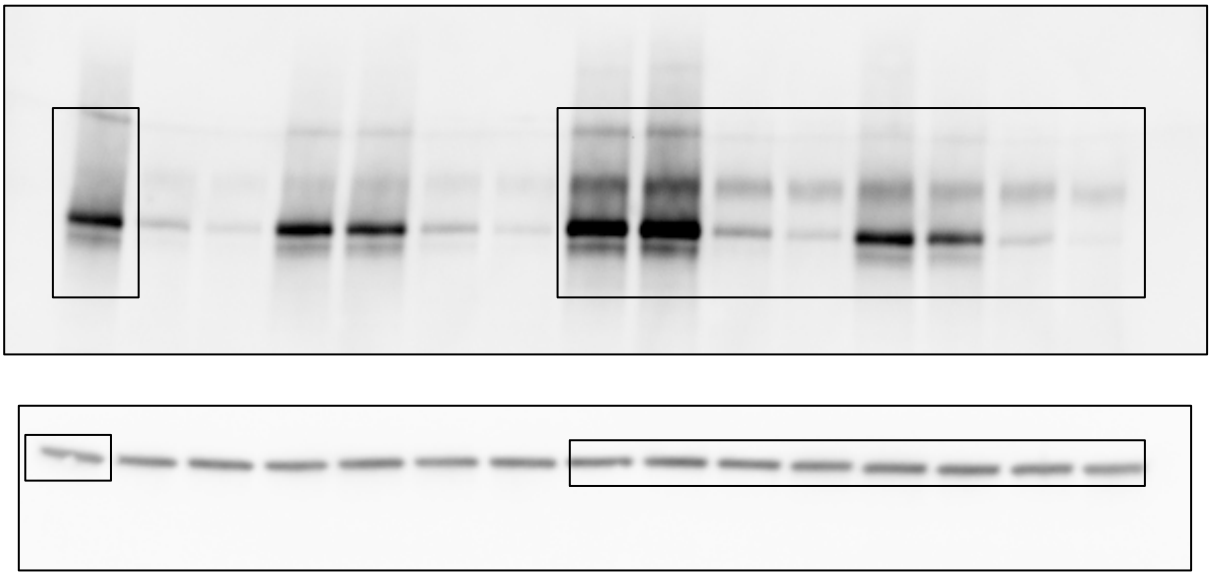

Figure 3A

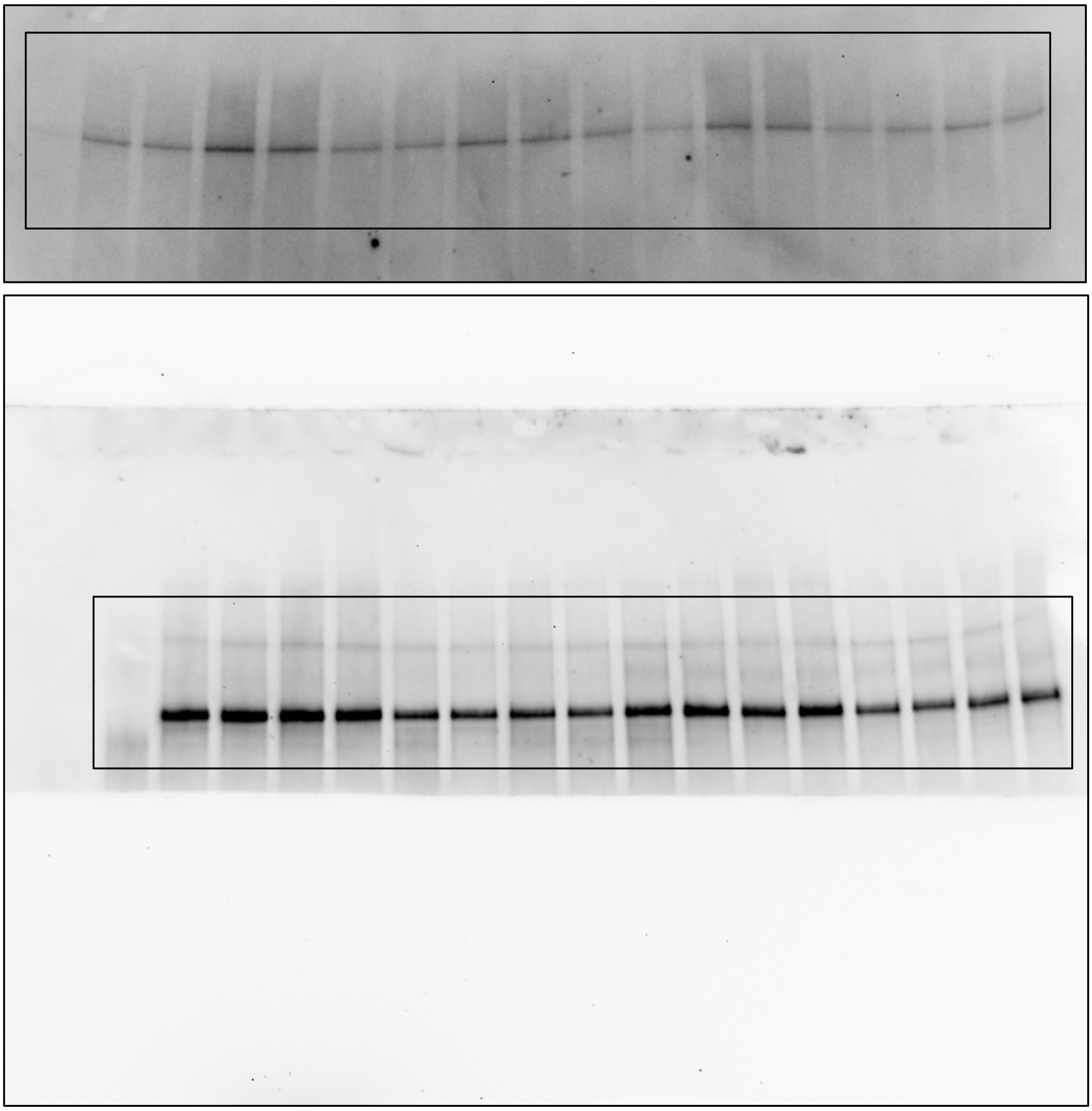

Figure 3B

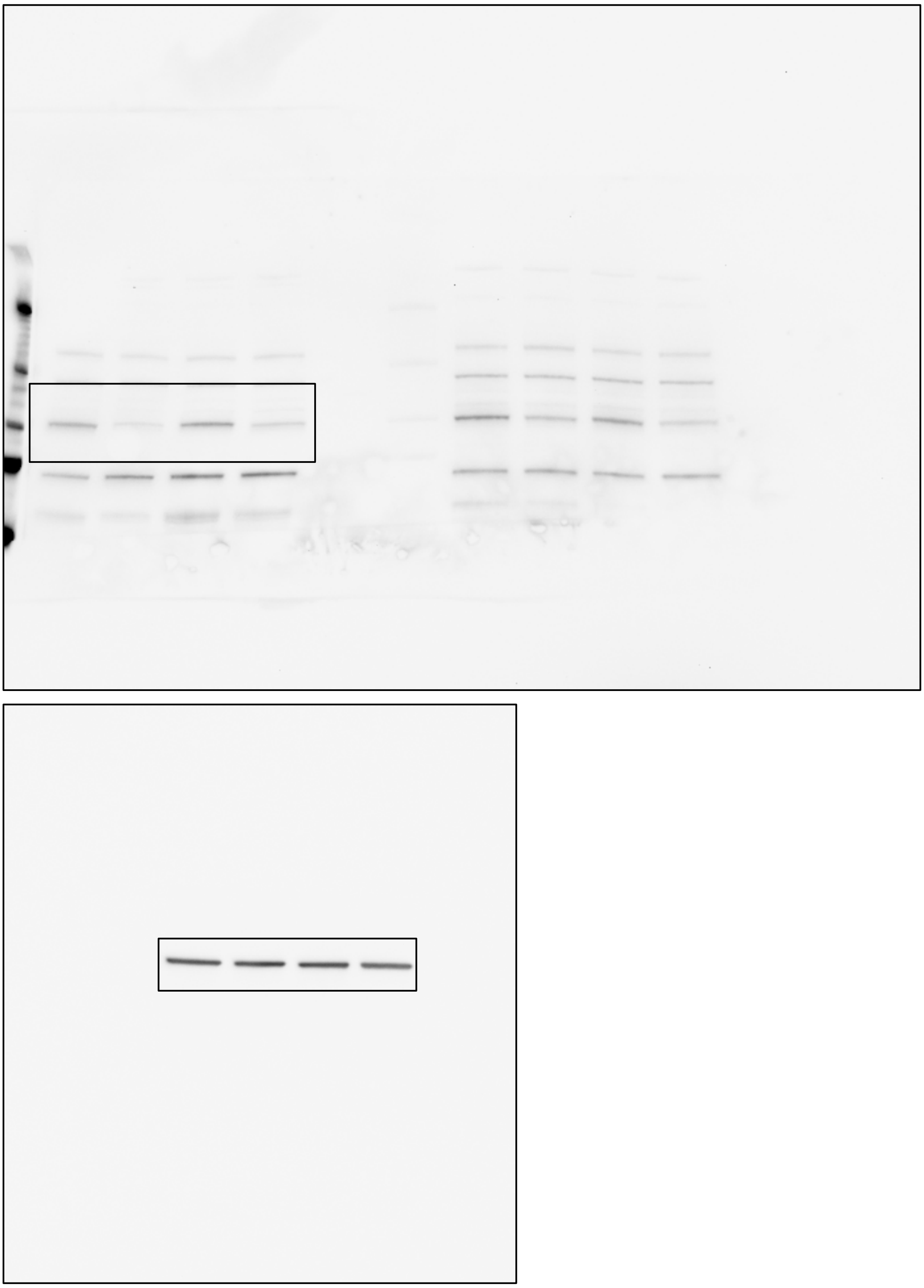

Figure 5B, left panel

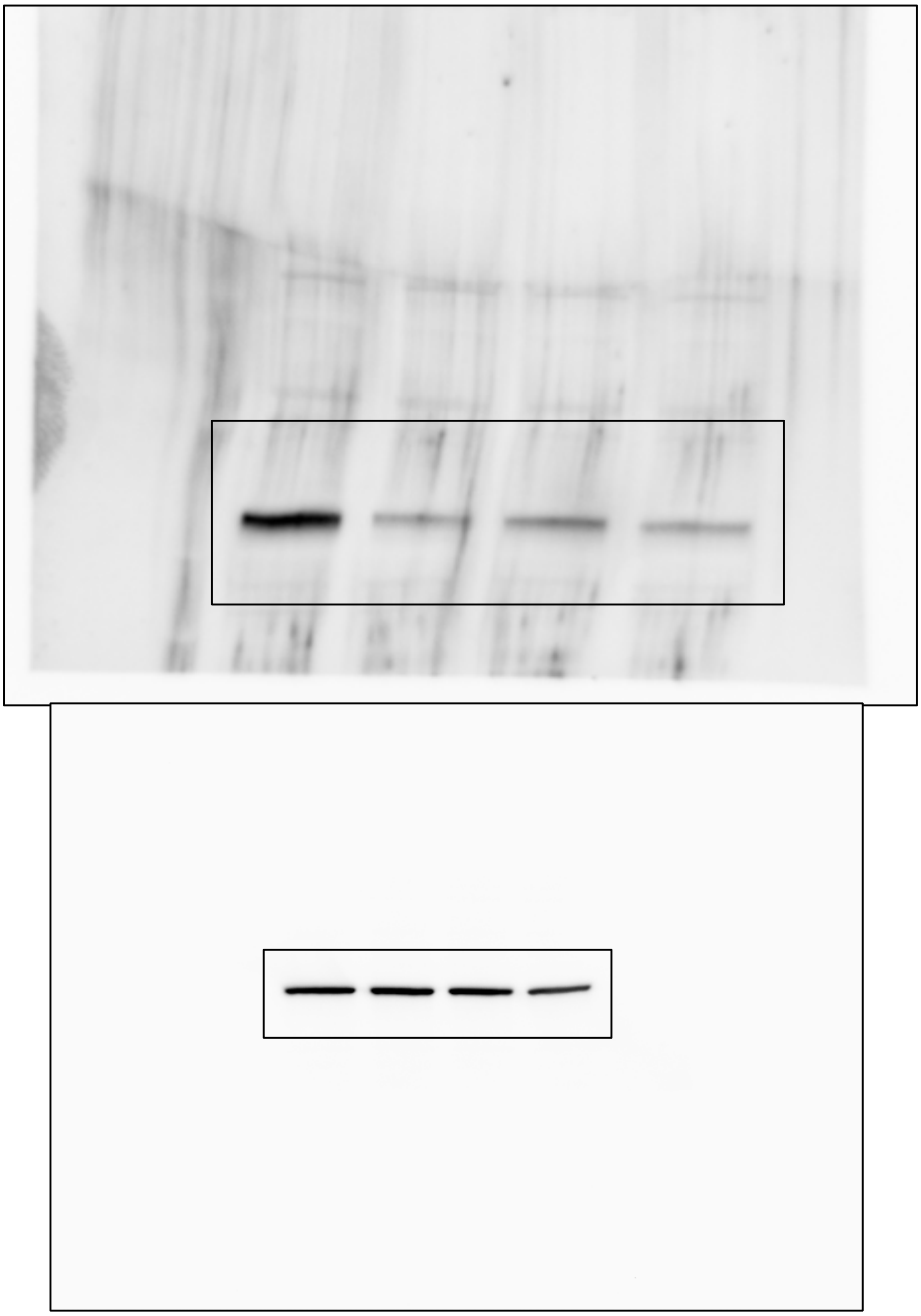

Figure 5B, right panel

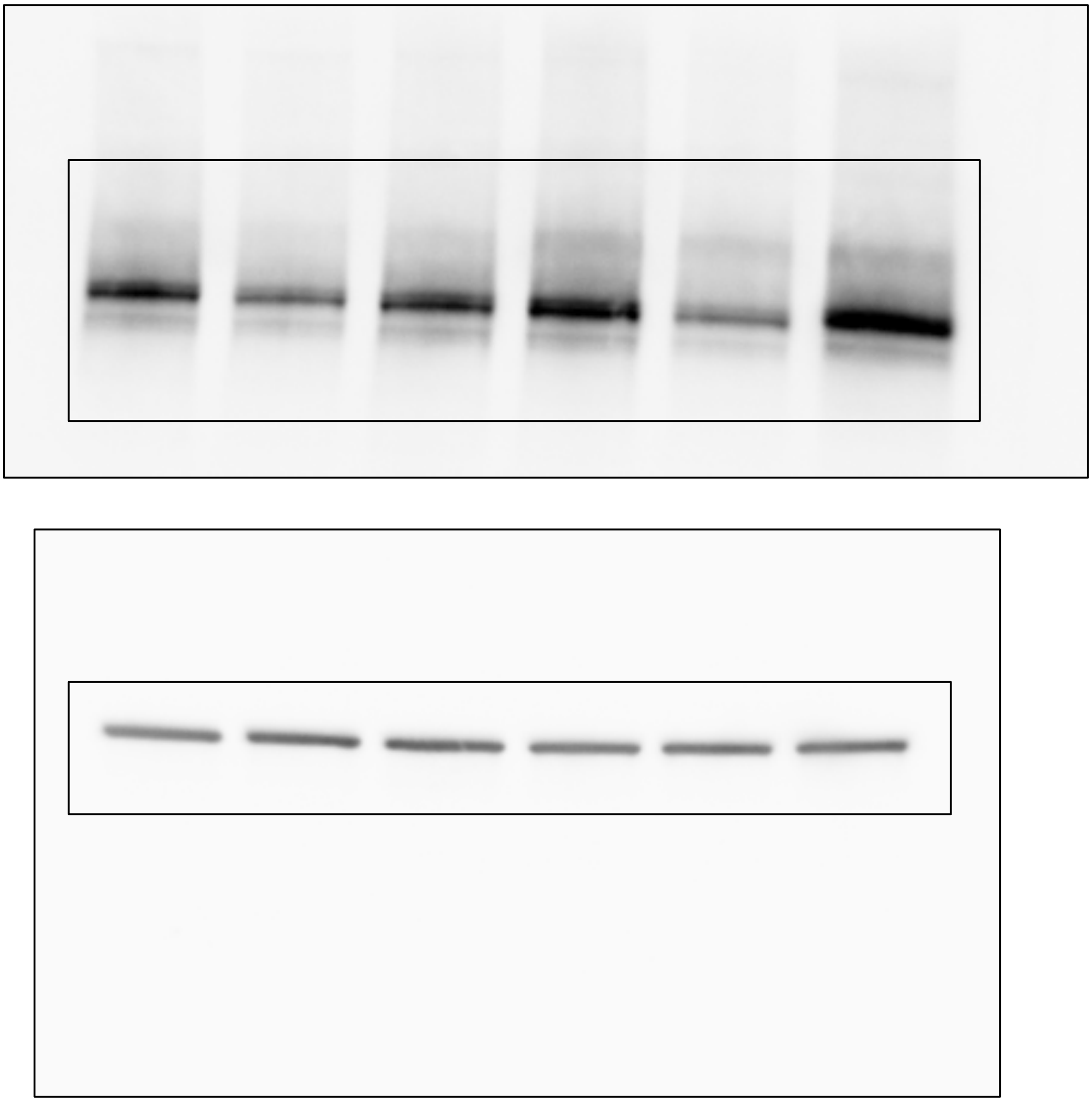

Figure 8B

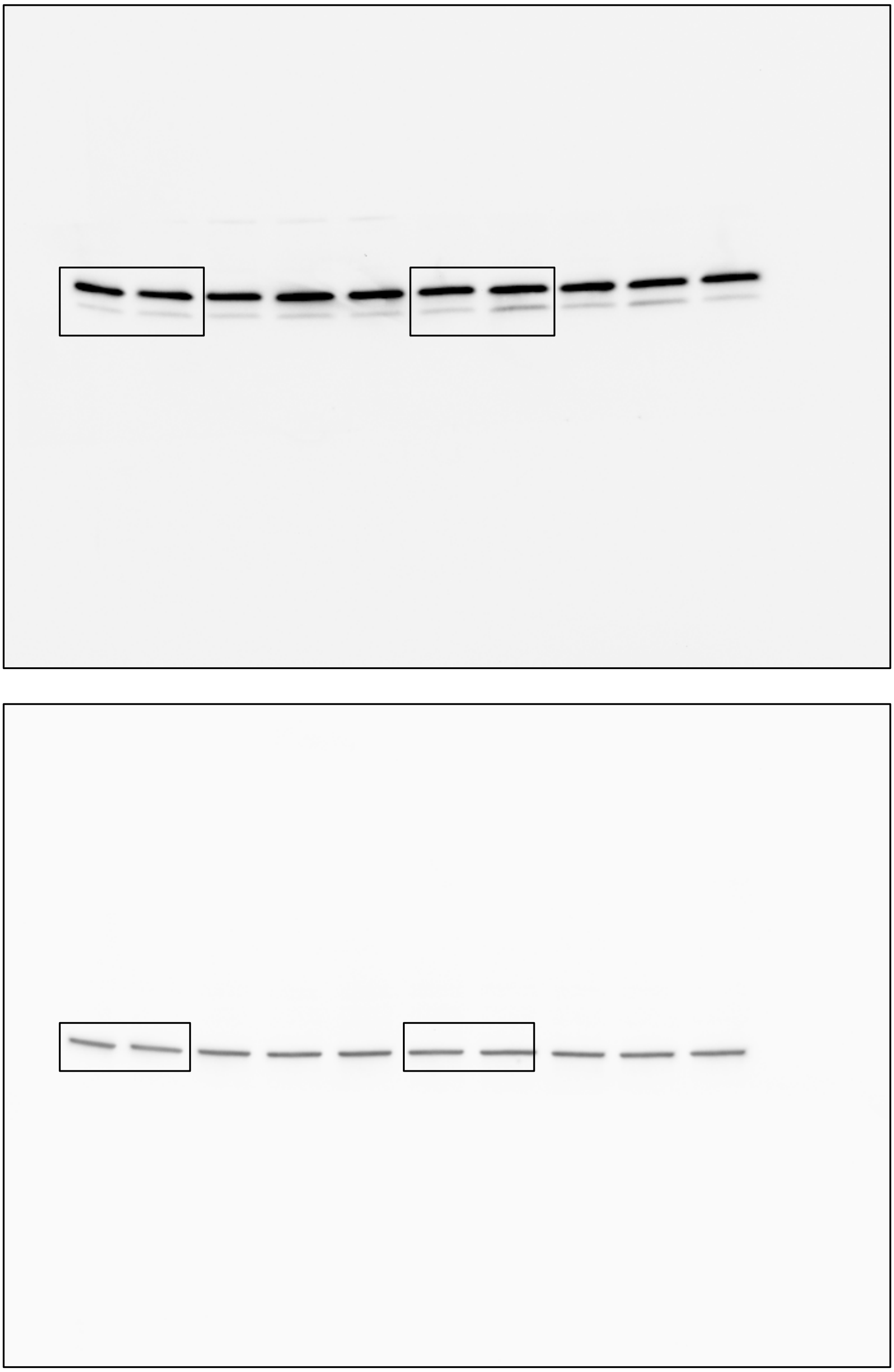

Figure 9B

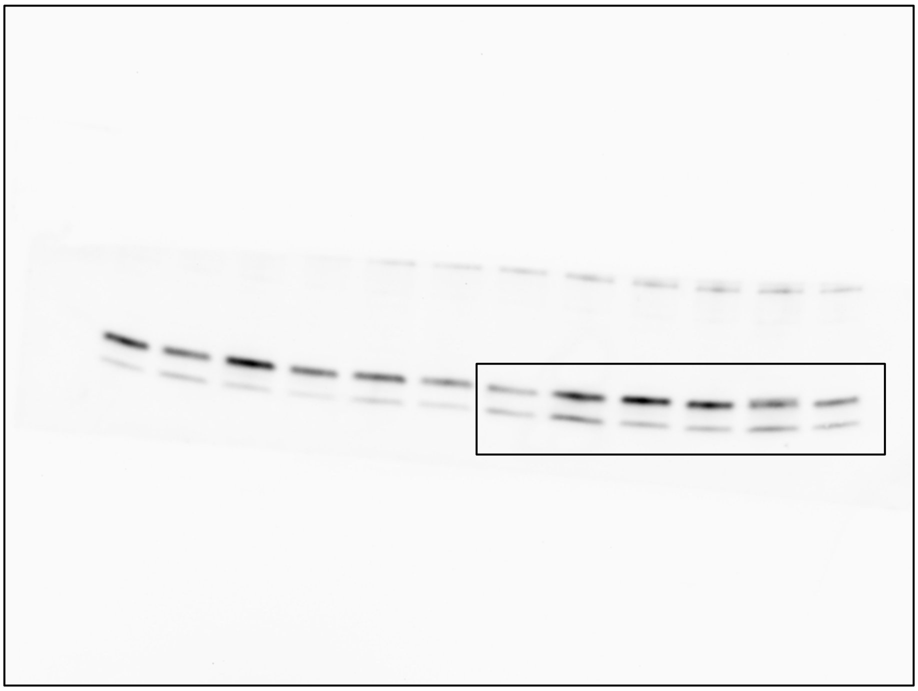

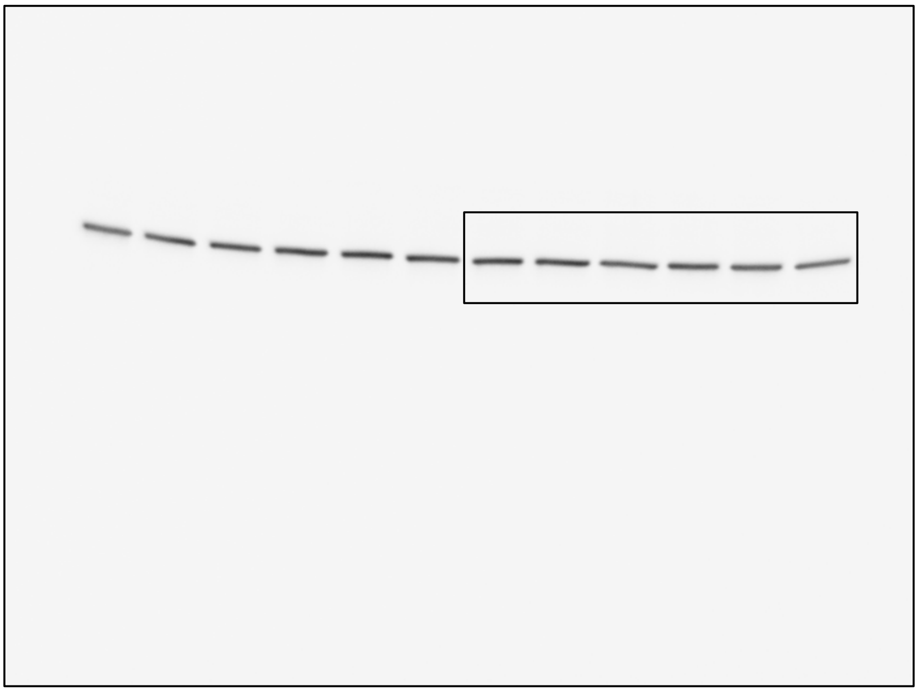

Figure 9C

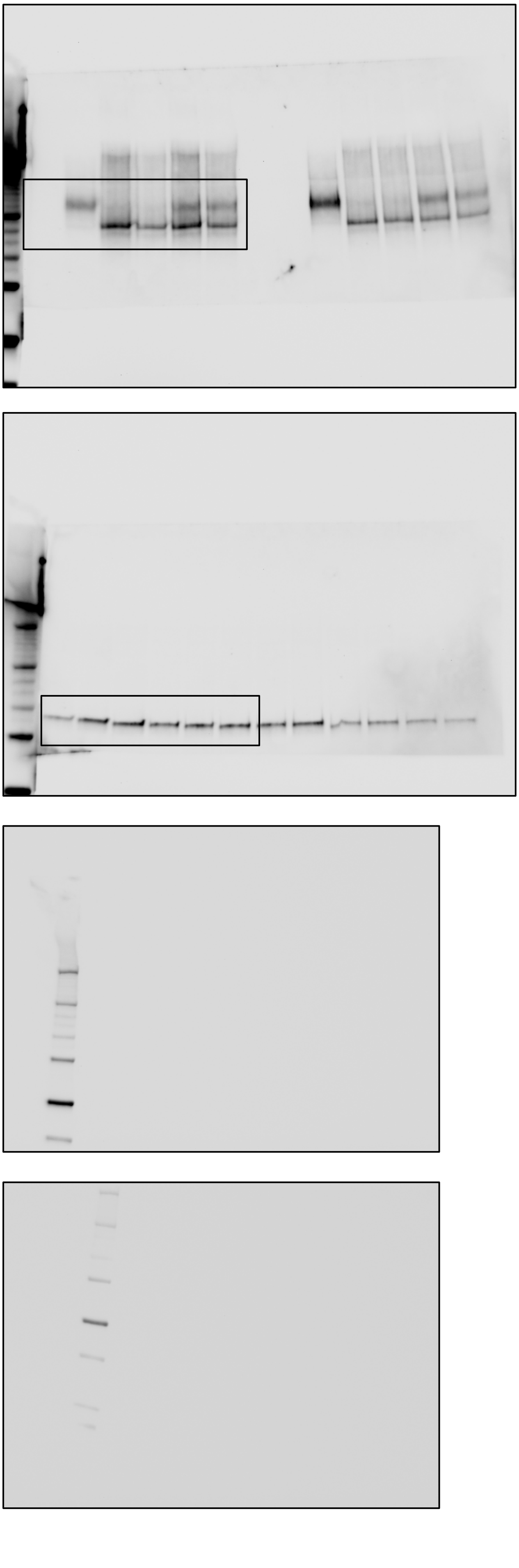

Suppl. Fig. 2
left panel

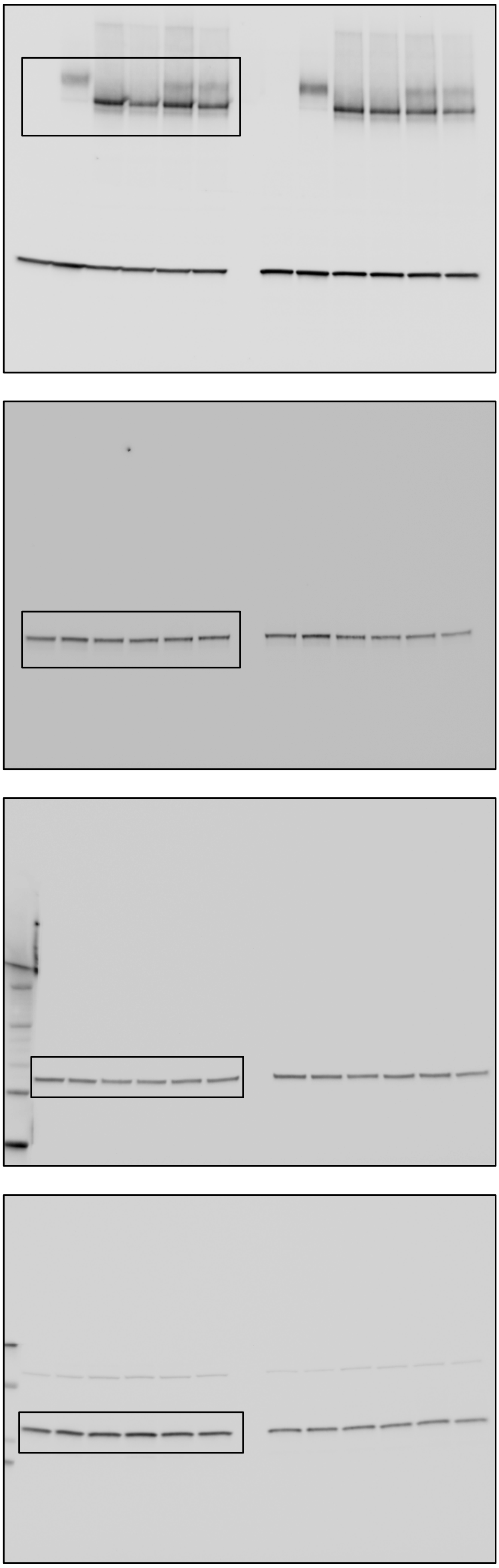

Suppl. Fig. 2
right panel

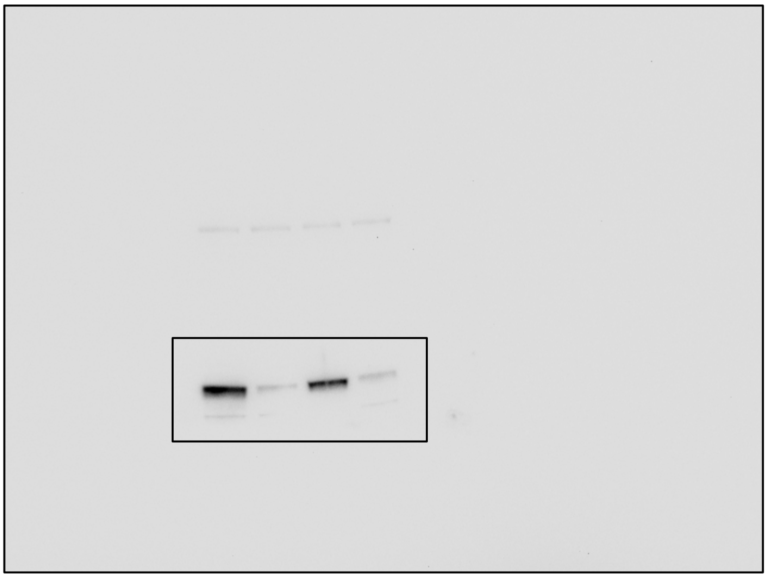

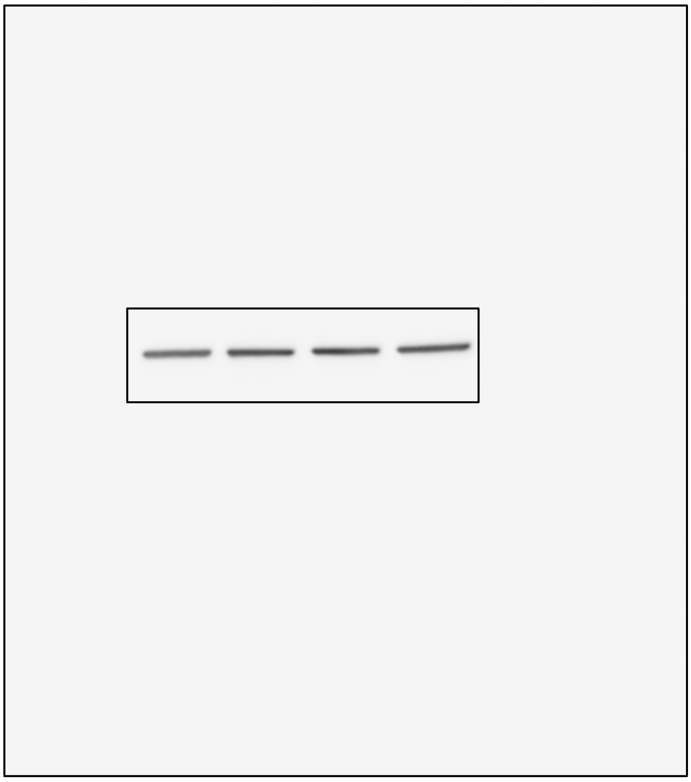

Suppl. Fig. 4C

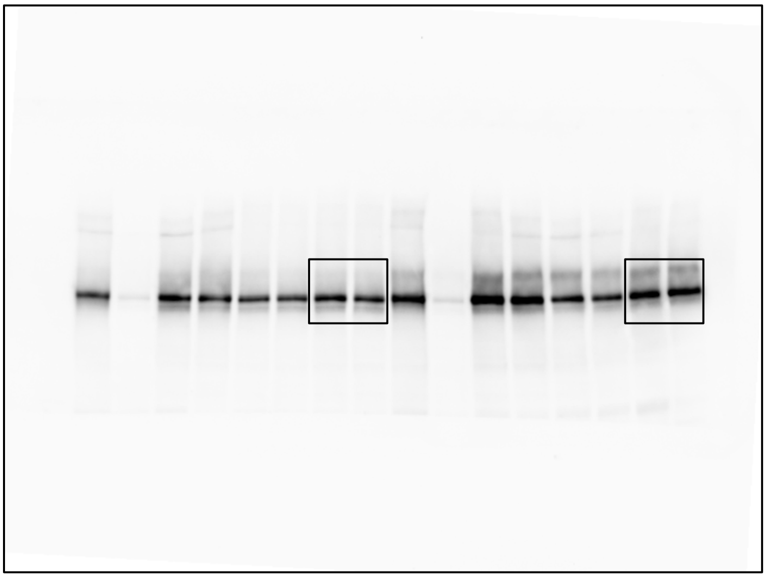

Suppl. Fig. 4D

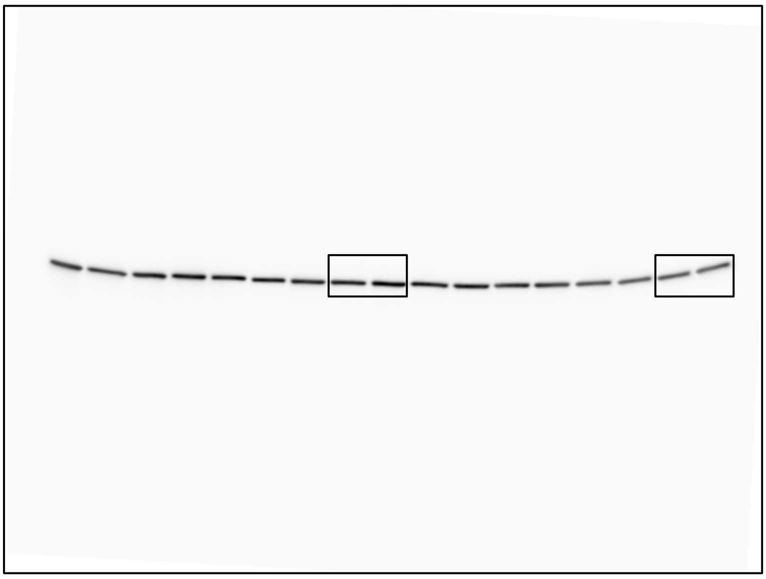

Supplement: Supplementary file 2 [file Data_Sheet_2.pdf]
